# Contrast weighted learning for robust optimal treatment rule estimation

**DOI:** 10.1002/sim.9574

**Published:** 2022-09-14

**Authors:** Xiaohan Guo, Ai Ni

**Affiliations:** ^1^ Division of Biostatistics, College of Public Health The Ohio State University Columbus Ohio

**Keywords:** contrast function, individualized treatment rule, ordinal outcome, personalized medicine, robustness

## Abstract

Personalized medicine aims to tailor medical decisions based on patient‐specific characteristics. Advances in data capturing techniques such as electronic health records dramatically increase the availability of comprehensive patient profiles, promoting the rapid development of optimal treatment rule (OTR) estimation methods. An archetypal OTR estimation approach is the outcome weighted learning, where OTR is determined under a weighted classification framework with clinical outcomes as the weights. Although outcome weighted learning has been extensively studied and extended, existing methods are susceptible to irregularities of outcome distributions such as outliers and heavy tails. Methods that involve modeling of the outcome are also sensitive to model misspecification. We propose a contrast weighted learning (CWL) framework that exploits the flexibility and robustness of contrast functions to enable robust OTR estimation for a wide range of clinical outcomes. The novel value function in CWL only depends on the pairwise contrast of clinical outcomes between patients irrespective of their distributional features and supports. The Fisher consistency and convergence rate of the estimated decision rule via CWL are established. We illustrate the superiority of the proposed method under finite samples using comprehensive simulation studies with ill‐distributed continuous outcomes and ordinal outcomes. We apply the CWL method to two datasets from clinical trials on idiopathic pulmonary fibrosis and COVID‐19 to demonstrate its real‐world application.

**Abbreviations**
CWL: contrast weighted learning
EARL: efficient augmentation and relaxation learning
OTR: optimal treatment rule
OWL: outcome weighted learning
RWL: residual weighted learning

## 1 INTRODUCTION

Motivated by the widely observed fact that medical treatments often exhibit heterogeneous effects across different patient subpopulations, personalized medicine that tailors treatments to individual patients has been a major biomedical and statistical research theme in recent years. The central idea of personalized medicine is to adapt an optimal treatment rule (OTR) to individual patients based on their personal characteristics such as demographics, biomarker measures, and environmental factors so that the overall treatment benefit on the patient population may be maximized. In addition, treating patients according to an OTR can potentially reduce the cost of clinical trials, avoid some predictable side effects, and increase patient's engagement and so forth.^1^

Existing statistical methods for OTR estimation can be generally categorized into two classes. One class relies on parametric or semi‐parametric modeling of the expected difference in the potential outcomes between treatments.^2^, ^3^ For example, the Q‐learning^4^ fits an $L^{1}$‐penalized linear regression model with emphasis on the interaction between treatment and covariates. Although easily implemented, these methods are vulnerable to model misspecification. The other class of methods aims at directly searching for a treatment assignment rule that maximizes a defined value function. A pioneer work in this class is the outcome weighted learning (OWL),^5^ which casts the OTR estimation into a weighted classification problem, opening the door to leveraging modern machine learning techniques for OTR estimation. There are, however, several limitations of OWL. First, it requires the outcome to be positive and the estimated OTR by OWL is affected by a simple shift of the outcome, making the estimate numerically unstable. Moreover, OWL tends to take the observed treatment assignment as their optimal treatment.^6^ To overcome these limitations, a group of weighted learning methods were proposed by positing a model for the outcome in combination with the weighted classification algorithm.^6^, ^7^, ^8^ In particular, residual weighted learning (RWL)^6^ replaces the outcome weights with residuals computed by subtracting a function of the covariates from the outcome and modifies the classification error to account for the sign of the residuals. More recently, Zhao et al^8^ develops an efficient augmentation and relaxation learning (EARL) method that endows the OWL with a doubly robust property by incorporating an outcome model into the value function. The OTR estimation methods using machine learning techniques are not limited to weighted learning. A tree‐based method^9^ is proposed to yield interpretable decision rule. Doubleday et al^10^ extends the single decision tree to random forest. An approach based on XGboost algorithm^11^ models the conditional mean of clinical outcome via additive regression trees.

Despite extensive studies, there are still limitations in the existing OTR methods. The consistency of Q‐learning critically depends on the correct specification of the outcome model. The finite sample performance of RWL and EARL is affected by the choice of the covariate function and the outcome model, respectively. Furthermore, since all these methods use the actual outcome values to construct value functions, they are likely to be vulnerable to heavy tails and outliers in the outcome distribution, which is common in clinical research. Many serum cytokine and chemokine measures are heavily skewed; the percent change from baseline tends to exhibit heavy tails and may even appear to be outlying if the baseline value is very small. A motivating example is the ACE‐IPF clinical trial investigating the efficacy of warfarin on patients with idiopathic pulmonary fibrosis (IPF).^12^ One outcome of interest is the percent change of lung forced vital capacity (FVC) from baseline to week 48, which shows some degree of left skewness and a possible outlier. A common strategy for handling heavy tails is to perform natural log or square root transformation on the original data. However, these transformations only work for positive outcomes and may not be sufficient to fully remove heavy tails. More complicated transformations such as the Box‐Cox transformation family^13^ are available to remedy these shortcomings, but they come at the cost of cumbersome interpretation. For outliers, it is generally not advisable to remove them from the dataset without strong clinical reasons, especially when the sample size is small. Thus, an OTR estimation method that is robust to ill‐behaved outcome distributions without ad‐hoc data processing is desirable.

Most of the OTR estimation methods were developed primarily for continuous outcomes. It can be challenging for them to accommodate other types of outcomes such as ordinal outcomes, especially for those methods that involve outcome modeling.^4^, ^6^, ^7^, ^8^ A motivating example is the ORCHID clinical trial evaluating the efficacy of hydroxychloroquine in patients hospitalized with COVID‐19.^14^ The primary outcome is a 7‐category ordinal scale measuring the severity of the disease.^15^ There are many choices of a link function for an ordinal outcome model such as adjacent category logit, cumulative logit, continuation‐ratio logit, or even an identity link function treating the ordinal outcome as a continuous variable. It can be difficult to determine which one is the best to use in practice, leading to possible model misspecification.

In this article, we propose a novel OTR estimation method that overcomes the aforementioned difficulties in the existing methods. The proposed method, named contrast weighted learning (CWL), uses contrasts of the outcome between pairs of subjects to construct the weights used in the weighted classification algorithm to estimate the OTR. Contrast functions have been used as an extension of group mean difference to measure the treatment effect in numerous clinical studies and demonstrate significant strengths in robustness and flexibility.^16^, ^17^ As an example, the celebrated Mann‐Whitney U test,^18^ a robust alternative to the two‐sample t‐test, is based on pairwise contrasts of measures between two comparison groups. A contrast h(x,y)=I(x≽y) (where I(·) is an 0‐1 indicator) can be defined as long as there exists an operator ≽ that measures the favorability of one variable over the other in the space that x and y reside. By choosing an appropriate contrast function h, one can achieve various levels of robustness to outcome distributions and adopt a wide range of outcome types. Tao and Wang^19^ proposed an adaptive contrast weighted OTR learning method that focuses on contrasts among potential outcomes conditional on covariates in a multi‐treatment setting. They only considered difference contrast so robustness is not of their concern. In comparison, the CWL methods proposed in this article construct marginal contrasts of outcomes between subjects whose covariates can be different, as will be made clear in the next section. Moreover, we consider contrast functions with different levels of robustness in the aim of developing a class of robust and flexible OTR methods.

The rest of this article is organized as follows. In Section 2, we define the framework of CWL and derive its risk function; In Section 3, the Fisher consistency and convergence rate of the estimated rule in CWL are established; Section 4 reports the extensive simulations on OTR estimation with continuous and ordinal outcomes while Section 5 demonstrates the application of CWL to two real clinical trial datasets. Finally, Section 6 offers a brief discussion of CWL in terms of applicability, computational performance, and potential extension.

## 2 METHODOLOGY

### 2.1 Outcome weighted learning

In randomized controlled trials or observational studies, suppose we observe the baseline covariates $X\in 𝒳\subset {\mathbb{R}}^{p}$, the binary treatment assignment A∈{−1,1}, and the clinical outcome, or reward, Y∈𝒴⊂ℝ, where a larger reward is more desirable. A treatment rule d:𝒳→{−1,1} is a decision rule that recommends treatment according to patients' baseline covariates. Let 𝒟 denote a class of decision rules under consideration. The value function associated with d is defined as

$$V_{O}(d)=E[Y|A=d(X)]=E\left[\frac{I(A=d(X))Y}{\pi (A,X)}\right],$$

where I(·) is an indicator function taking values 0 and 1 and π(A,X)=Pr(A|X) is the propensity score of treatment assignment. The optimal decision rule $d^{*}$ is obtained by maximizing $V_{O}(d)$, or equivalently minimizing the risk function E[I(A≠d(X))Y/π(A,X)]. Given i.i.d. observed data $\left(Y_{i},A_{i},X_{i}\right)$, i=1,…,n, $d^{*}$ can be estimated by

(1) $$\hat{d}=\underset{d\in 𝒟}{\text{arg min}}\;\frac{1}{n}\overset{n}{\underset{i=1}{\sum }}\frac{I(A_{i}\neq d(X_{i}))Y_{i}}{\pi (A_{i},X_{i})}.$$

In randomized controlled trials, π(A,X) is known by design; in observational studies, a propensity score model is usually fitted to estimate it.

In their seminal work, Zhao et al^5^ treated the optimization problem in (1) as a weighted classification problem with weight Y/π(A,X), hence the name outcome weighted learning. OWL greatly advanced the field of OTR by leveraging the power of machine learning techniques to estimate the OTR. Many new OTR methods have been developed since the inception of the OWL. However, all these methods use the actual values of Y to construct weights, making them potentially sensitive to the outcome distribution. The methods that involve outcome models are also susceptible to model misspecification, especially for non‐continuous outcome types such as ordinal outcomes.

### 2.2 Contrast weighted learning (CWL)

Contrast functions have been widely used as measures of treatment effect in clinical studies. In our proposed method, a contrast function h:𝒴×𝒴→ℝ is defined for any two subjects to measure the relative favorability of their outcomes. The contrast function is assumed to satisfy the following regularity conditions for any x,y∈ℝ,

(i) h(x,x)=0;
(ii) h(x,y)>0 whenever x>y;
(iii) h(x,y)=−h(y,x);
(iv) For any fixed y, h(x,y) is an increasing function of x∈ℝ.

Various contrast functions can be used in CWL. The most common one is difference: $h(Y_{i},Y_{j})=Y_{i}-Y_{j}$. If $Y\in {\mathbb{R}}^{+}$ with a skewed distribution, the log ratio contrast $h(Y_{i},Y_{j})=\log(Y_{i}/Y_{j})$ may be preferred. When Y is susceptible to outliers, the win indicator $h(Y_{i},Y_{j})=\text{sgn}(Y_{i}-Y_{j})$ is usually more robust than the difference or the ratio, where sgn(x)=1, if x>0; sgn(x)=0, if x=0; sgn(x)=−1, if x<0.

Given a contrast function h(·,·) and i.i.d. observations $\left(Y_{i},A_{i},X_{i}\right)$, i=1,…,n, define the contrast weighted value function as

(2) $$V(d)=E\left[\frac{W_{i}(d)(1-W_{j}(d))h(Y_{i},Y_{j})}{\pi (A_{i},X_{i})\pi (A_{j},X_{j})}\right],$$

where i,j are the indexes of two arbitrary subjects in data and $W_{i}(d)=I(A_{i}=d(X_{i}))$, for any i=1,…,n, is a compliance indicator of whether a subject follows the rule d under the observed assignments. Since the observations are i.i.d., subjects i and j in the value function are exchangeable. The expectation is with respect to the joint distribution of $\left(Y_{i},A_{i},X_{i}\right)$ and $\left(Y_{j},A_{j},X_{j}\right)$. Essentially, the value function is the expected favorability in the outcome of a subject who follows the rule d vs the outcome of a subject defying d, where the favorability is measured by an inverse probability weighted contrast $h(Y_{i},Y_{j})/[\pi (A_{i},X_{i})\pi (A_{j},X_{j})]$. Since we treat each pair as a unit in our value function, the inverse probability weights for subjects i,j are multiplied to form the weight for pair (i,j). Using contrast functions and paired inverse probability weighting, Mao^16^ proposed U‐statistics based estimators for treatment causal effect. Our value function follows the similar idea, but the comparison groups of interest in the CWL are defined by the compliance indicator $W_{i}(d)$, which depends on $A_{i},X_{i}$, and d.

A decision rule d(X) can be represented as sgn(f(X)) for a measurable function f:𝒳→ℝ. (Here we define sgn(0)=1.) Then, $W_{i}(d)=I(A_{i}f(X_{i})\geq 0)$ and the value function V(d) can be rewritten as V(f). The optimal decision rule $d^{*}(X)=\text{sgn}(f^{*}(X))$, where $f^{*}={\text{arg max}}_{f}V(f)$. Moreover, maximizing V(f) with respect to f is equivalent to minimizing a risk function R(f)=c−V(f), for any c that does not depends on f. After some derivations (see Appendix A for details), we find that the optimal decision function $f^{*}$ is the minimizer of the following risk function:

(3) $$R(f)=\frac{1}{2}E\{[I(\text{sgn}(h(Y_{i},Y_{j}))A_{i}f(X_{i})<0)+I(\text{sgn}(h(Y_{i},Y_{j}))A_{j}f(X_{j}))\geq 0)]\frac{\left|h(Y_{i},Y_{j})\right|}{\pi (A_{i},X_{i})\pi (A_{j},X_{j})}\}.$$

In the above risk function, we separate sign and scale of the contrast function $h(Y_{i},Y_{j})$ to guarantee that the contrast‐based weight $\left|h(Y_{i},Y_{j})|/[\pi (A_{i},X_{i})\pi (A_{j},X_{j})\right]$ is non‐negative. The sign of the contrast is incorporated into the 0‐1 loss functions. Since it is difficult to minimize R(f) with the 0‐1 loss due to its discontinuity and nonconvexity, we approximate it with the hinge loss ϕ(t)=max{1−t,0}. Define the surrogate ϕ‐risk function as

$$R_{\phi }(f)=\frac{1}{2}E\{[\phi (\text{sgn}(h(Y_{i},Y_{j}))A_{i}f(X_{i}))+\phi (-\text{sgn}(h(Y_{i},Y_{j}))A_{j}f(X_{j}))]\frac{\left|h(Y_{i},Y_{j})\right|}{\pi (A_{i},X_{i})\pi (A_{j},X_{j})}\}.$$

Given observed data $\left(Y_{i},A_{i},X_{i}\right)$, i=1,…,n, the empirical risk function is defined as

(4) 12n2−1∑i<j{[ϕ(sgn(h(Yi,Yj))Aif(Xi))+ϕ(−sgn(h(Yi,Yj))Ajf(Xj))]|h(Yi,Yj)|π(Ai,Xi)π(Aj,Xj)}.

After some algebra (see Appendix A for details), we find that minimizing (4) with respect to f is equivalent to minimizing the following empirical risk function:

(5) $$\frac{1}{n}\overset{n}{\underset{i=1}{\sum }}\phi ({\tilde{A}}_{i}f(X_{i}))\frac{\left|C_{i,n}^{h}\right|}{\pi (A_{i},X_{i})},$$

where $C_{i,n}^{h}={\left(n-1\right)}^{-1}\sum_{j\neq i}h(Y_{i},Y_{j})/\pi (A_{j},X_{j})$ and ${\tilde{A}}_{i}=A_{i}\text{sgn}(C_{i,n}^{h})$ is a pseudo treatment assignment depending on the sign of $C_{i,n}^{h}$. Thus, the empirical risk function of the CWL takes on a similar form as that of the OWL^5^ with pseudo treatment ${\tilde{A}}_{i}$ and weight $\left|C_{i,n}^{h}|/\pi (A_{i},X_{i}\right)$. Compared to the OWL, our method defines a novel, contrast‐based weight that is flexible to accommodate a wide variety of outcome distributions and offers robustness. $C_{i,n}^{h}$ can be interpreted as the average contrast of $Y_{i}$ over the outcomes of other subjects in the sample (adjusting for treatment propensity). Heuristically, the sign of $C_{i,n}^{h}$ indicates whether $Y_{i}$ is above or below the average level in the sample. If a subject has an outcome lower than the average level (ie, $\text{sgn}(C_{i,n}^{h})=-1$), its observed treatment assignment is flipped so that a decision that follows the original observed treatment assignment will incur loss and therefore be discouraged.

We consider the estimation of the decision function f as a constant plus the function in reproducing kernel Hilbert space (RKHS) ${ℋ}_{k}$ with kernel function k:𝒳×𝒳→ℝ and norm $\|\cdot {\|}_{k}$. Then f(·) takes the form $\sum_{i=1}^{n}\alpha_{i}{\tilde{A}}_{i}k(\cdot ,X_{i})+\alpha_{0}$, where $\alpha_{i}$, i=0,…,n are coefficients to be estimated. To prevent overfitting, we adopt a regularization term to control the complexity of f. Thus, the optimal decision function $f^{*}$ is estimated by

(6) $${\hat{f}}_{n}=\underset{f\in {ℋ}_{k}}{\text{arg min}}\frac{1}{n}\overset{n}{\underset{j=1}{\sum }}\phi ({\tilde{A}}_{i}f(X_{i}))\frac{\left|C_{i,n}^{h}\right|}{\pi (A_{i},X_{i})}+\lambda_{n}\|f{\|}_{k}^{2},$$

where $\lambda_{n}$ is a tuning parameter. The optimization problem in (6) can be solved by fitting a weighted support vector machine (SVM) as in Zhao et al^5^ with class label ${\tilde{A}}_{i}$ and weight $\left|C_{i,n}^{h}|/\pi (A_{i},X_{i}\right)$. The weighted support vector machine can be formulated as a dual problem and then solved by quadratic programming algorithm. See Web Appendix 2.1 in Supporting Information for details.

## 3 THEORETICAL PROPERTIES OF CWL

In this section, we establish theoretical properties of contrast weighted learning, including Fisher consistency and convergence rate of the optimal empirical risk when using RKHS generated by a Gaussian kernel.

### 3.1 Fisher consistency

Theorem 1 (Fisher consistency) *Given a contrast function* h *that satisfies conditions (i) to (iv), for any measurable function* f *, if* $\tilde{f}$ *minimizes* $R_{\phi }(f)$ *, then the optimal treatment rule* $d^{*}=\text{sgn}(\tilde{f}(X))$.

The proof of Theorem 1 is included in Appendix B.

### 3.2 Convergence rate

Define $R^{*}=\inf_{f}\{R(f)|f:𝒳\to \mathbb{R}\;\text{is measurable}\}$. To derive the convergence rate of $R({\hat{f}}_{n})$ to $R^{*}$, we consider the RKHS generated by Gaussian kernel $k(x,y)=\exp(-\sigma_{n}^{2}\|x-y{\|}^{2})$, for $x,y\in {\mathbb{R}}^{p}$, $\sigma_{n}>0$. Since it is impossible to establish a uniform convergence rate of $R({\hat{f}}_{n})$ for an arbitrary distribution of (X,A,Y),^6^, ^20^ we assume the geometric noise^21^ on the distribution of (X,A,Y). Let $Q_{ij}^{h}=h(Y_{i},Y_{j})/\pi (A_{j},X_{j})$. Define

$$\eta (x_{i})=\frac{E(Q_{ij}^{h}|A_{i}=1,X_{i}=x_{i})-E(Q_{ij}^{h}|A_{i}=-1,X_{i}=x_{i})}{2}+\frac{1}{2},$$

where the conditional expectations are with respect to the distribution of $\left(Y_{i},Y_{j},A_{j},X_{j}\right)$ given $X_{i},A_{i}$. $E(Q_{ij}^{h}|A_{i}=a,X_{i}=x_{i})$ is the expected contrast of outcome of subject i with $A_{i}=a$ vs the outcome of a random subject j and the decision boundary is $2\eta (x_{i})-1$. Let ${𝒳}^{+}=\{x\in 𝒳:2\eta (x)-1\geq 0\}$ and ${𝒳}^{-}=\{x\in 𝒳:2\eta (x)-1<0\}$. Define a distance function to the boundary: $\Delta (x)=\tilde{d}(x,{𝒳}^{+})$ if $x\in {𝒳}^{+}$; otherwise, $\Delta (x)=\tilde{d}(x,{𝒳}^{-})$, where $\tilde{d}(x,𝒪)$ is the Euclidean distance from a set 𝒪. The distribution of (X,A,Y) has a geometric noise exponent 0<q<∞, if there exists a constant C>0 such that

(7) $$E\left[\exp\left(\frac{-\Delta {\left(X_{i}\right)}^{2}}{t}\right)|2\eta (X_{i})-1|\right]\leq Ct^{qp/2},\;t>0.$$

Theorem 2 (Convergence rate) *Assume that* $\left(X_{i},A_{i},Y_{i}\right)$ *has geometric noise exponent* q>0 *satisfying (* *7* *) and let* $\sigma_{n}=\lambda_{n}^{-1/(q+1)p}$ *for* $\lambda_{n}\to 0$ *and* $n\lambda_{n}\to \infty$ *, then for any* δ>0 *,* 0<ν<2 *, there exists a constant* μ(δ,ν,p) *such that for all* τ≥1 *,*

$$P(R({\hat{f}}_{n})\leq R^{*}+\epsilon )\geq 1-e^{-\tau },$$

*where*

$$\epsilon =\mu (\delta ,\nu ,p)\left[\lambda_{n}^{-\frac{2}{2+\nu }+\frac{\left(2-\nu )(1+\delta \right)}{\left(2+\nu )(1+q\right)}}n^{\frac{-2}{2+\nu }}+\frac{\tau }{n\lambda_{n}}+\lambda_{n}^{\frac{q}{q+1}}+n^{-\frac{1}{2}}\right].$$

*In particular, given the optimal choice of tuning parameter* $\lambda_{n}=n^{-2(1+q)/[(4+\nu )q+2+(2-\nu )(1+\delta )]}$ *, the optimal convergence rate for the risk is* $R({\hat{f}}_{n})-R^{*}=O_{p}(n^{-2q/[(4+\nu )q+2+(2-\nu )(1+\delta )]})$.

Theorem 2 shows that the optimal convergence rate of $R({\hat{f}}_{n})$ under the CWL is the same as that under the OWL.^5^ The proof of Theorem 2 basically follows the idea of Steinwart and Scovel^21^ and Zhao et al.^5^ The excess risk of R(f) is bounded by the excess ϕ‐risk, then the convergence rate of excess ϕ‐risk is derived by adapting the theoretical results for (weighted) SVM. Importantly, the proposed CWL has a risk function in the form of U‐statistics, so the idea of Hoeffding's decomposition^22^ is implemented to adjust for the dependence introduced by the pairwise contrast function. The detailed proof is included in the Web Appendix 1.2.

## 4 SIMULATION STUDIES

We conduct extensive simulations under observational study design to compare the finite sample performance of the proposed contrast weighted learning to several existing methods of OTR estimation, including Q‐learning,^4^ OWL,^5^ RWL,^6^ and EARL.^8^ Two types of outcomes (continuous and ordinal) are considered.

### 4.1 Continuous outcome

Define the data generating model of a continuous outcome $Y_{i}$ (i=1,…,n) as

$$Y_{i}=\mu (X_{i})+A_{i}T(X_{i})+Z_{i}O_{i}+e_{i},$$

where $A_{i}T(X_{i})$ models the interaction between treatment and covariates and determines the optimal decision boundary, $\mu (X_{i})$ models the main effect of covariates, $O_{i}∼N(\mu_{O},3^{2})$ represents the outlier, $Z_{i}$ is an indicator function for the presence of outlier, and $e_{i}$ is a random error with $E(e_{i})=0$. We consider the following six scenarios:

(1.a) Linear $\mu (X_{i})$ and $T(X_{i})$, no outlier, and $e_{i}$ follows a normal distribution;
(1.b) Nonlinear $\mu (X_{i})$ and $T(X_{i})$, no outlier, and $e_{i}$ follows a normal distribution;
(1.c) Linear $\mu (X_{i})$ and $T(X_{i})$, outliers present, and $e_{i}$ follows a normal distribution;
(1.d) Nonlinear $\mu (X_{i})$ and $T(X_{i})$, outliers present, and $e_{i}$ follows a normal distribution;
(1.e) Linear $\mu (X_{i})$ and $T(X_{i})$, no outlier, and $e_{i}$ follows a heavy‐tailed distribution;
(1.f) Nonlinear $\mu (X_{i})$ and $T(X_{i})$, no outlier, and $e_{i}$ follows a heavy‐tailed distribution.

We assume the jth covariate of the ith subject $X_{ij}\overset{i.i.d.}{∼}U(-1,1)$ for i=1,…,n, j=1,…,p. In linear decision boundary settings, we set p=10, $T(X_{i})=0.5+X_{i1}-X_{i2}$, and $\mu (X_{i})=2X_{i3}-X_{i4}$; In nonlinear decision boundary settings, we set p=5, $T(X_{i})=5(0.5-X_{i1}^{2}-X_{i2}^{2})$, and $\mu (X_{i})=X_{i3}^{2}-X_{i4}^{2}$. The mean of the outlier term $O_{i}$ is set to $\mu_{O}=15$ in all scenarios. In scenarios without outlier, $Pr(Z_{i}=1)=0$; in scenarios with outlier, 3% of $Z_{i}$ (i=1,…,n) are randomly selected to be 1 and others are 0. Under normal error, $e_{i}\overset{i.i.d.}{∼}N(0,1)$; under heavy‐tailed error, $e_{i}=e_{i}^{\prime }-\exp(\sigma_{\log}^{2}/2)$, where $e_{i}^{\prime }$ follows a log normal distribution with location parameter 0 and scale parameter $\sigma_{\log}=1.5$. Since $E(e_{i}^{\prime })=\exp(\sigma_{\log}^{2}/2)$, $E(e_{i})=0$. In all scenarios, the true propensity score model is $\text{logit}[P(A_{i}=1|X_{i})]=0.2X_{i1}-0.2X_{i3}+0.2X_{i5},\;i=1,...,n$. Following Zhao et al,^8^ we estimate the propensity score by an $L^{1}$‐penalized logistic regression using $X_{i}$, where the tuning parameter is selected via 10‐fold cross‐validation.

In Q‐learning, an $L^{1}$‐penalized linear regression model is fitted with main effects of $X_{i}$, A, and their pairwise interactions. Thus, the regression model is correctly specified in linear boundary settings but misspecified in nonlinear boundary settings. In OWL, outcomes are shifted by $Y_{i}-\min(Y_{i})$ to avoid negative weights. In RWL, we fit a linear regression model that includes all the main effects of covariates to estimate the residuals. The choice of main effect model in RWL can be flexible. We also use random forest for the main effect model but do not find any significant difference in performance (see Supporting Information). In EARL, the outcome model is identical to that used in the Q‐learning. In CWL, we consider three types of contrast function: (1) Difference: $h_{1}(x,y)=x-y$; (2) Truncated difference: $h_{2}(x,y;t)=\text{sgn}(x-y)\cdot \min(|x-y|,t)$, where t is a constant; (3) Win indicator: $h_{3}(x,y)=\text{sgn}(x-y)$. We define a data‐driven t as $\min(|Q_{0.25}-1.5\times IQR|,|Q_{0.75}+1.5\times IQR|)$, where $Q_{\alpha }$ is the sample α‐quantile of $h(Y_{i},Y_{j})$ for all i,j and $IQR=Q_{0.75}-Q_{0.25}$. Among the three contrast functions, $h_{1}$ is the least robust as it uses the actual values of the outcome; $h_{3}$ is the most robust as it only depends on the ranking of outcome values; $h_{2}$ lies in between $h_{1}$ and $h_{3}$ in terms of robustness. For all methods except Q‐learning, we estimate the OTR using linear kernels for linear $T(X_{i})$ scenarios and Gaussian kernels for nonlinear $T(X_{i})$ scenarios. We use R package DynTxRegime ^23^ to implement Q‐learning, RWL, EARL and package WeightSVM ^24^ to implement OWL and CWL. The tuning parameters are selected by 4‐fold cross‐validation in all methods.

For each scenario, we consider sample sizes 50, 150, and 500. For each sample size, 400 simulation replications are conducted. In each replication, we also generate a large validation dataset of size 50 000 where each subject has a pair of potential outcomes $Y_{i}^{\left(1\right)}$ and $Y_{i}^{\left(-1\right)}$. $Y_{i}^{\left(a\right)}$ are generated by the same model for training data but no outliers are included. That is, $Y_{i}^{\left(a\right)}=\mu (X_{i})+a\cdot T(X_{i})+e_{i}$, for a=−1,1. Although the value function is defined differently across OTR estimation methods, we calculate the value of an estimated rule $\hat{d}$ as $5\times 10^{-4}\sum_{i=1}^{50000}Y_{i}^{\left(\hat{d}(X_{i})\right)}$ for all methods to make it comparable across methods.

**FIGURE 1:**
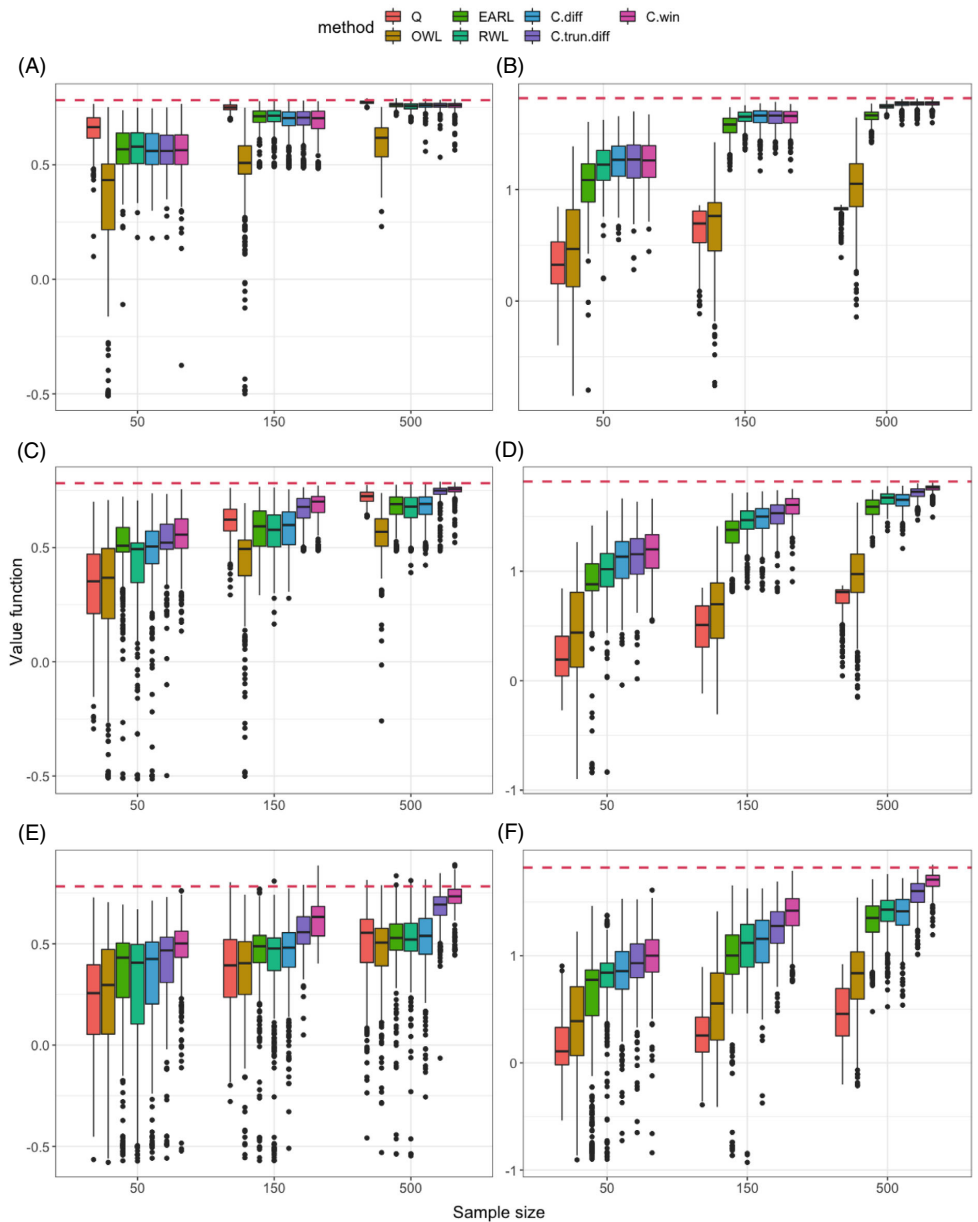
Testing value functions in 400 replications for six scenarios with continuous outcomes. The seven methods we compare (from left to right in plots) are: Q‐learning (Q), outcome weighted learning (OWL), efficient augmentation and relaxation learning (EARL), residual weighted learning (RWL), and contrast weighted learning using difference contrast (C.diff), truncated difference contrast (C.trun.diff), and win indicator contrast(C.win). The dashed horizontal line represents the theoretical optimal value. (A) Linear, normal; (B) nonlinear, normal; (C) linear, outlier; (D) nonlinear, outlier; (E) linear, heavy‐tailed; (F) nonlinear, heavy‐tailed

The values of the seven OTR estimation methods under the six scenarios are summarized by boxplots in Figure 1. When neither outlier nor skewness presents in the outcome (1.a and 1.b), CWL methods give comparable values to those of the best performer among existing methods. When outliers present in the outcome, CWL with win indicator has higher values than all existing methods under a linear boundary (1.c) and its advantage becomes more prominent under a nonlinear boundary (1.d). Similar observations can be made under skewed outcome distribution (1.e and 1.f). As expected, the Q‐learning works best under the conventional situation with a linear boundary (1.a) where its parametric model is correctly specified. Under nonlinear boundaries, its performance can be much worse due to model misspecification (1.b, 1.d, and 1.f). And the skewness of outcomes can also challenge the performance of Q‐learning as scenario (1.e) shows. The values of OWL are consistently low, likely due to its ad‐hoc shifting of outcomes and the fact that the outcome weights are not centered around zero as in RWL and CWL, making it especially sensitive to outliers and skewness in the outcome distribution. Among the three CWL methods, the win indicator contrast performs best, closely followed by the truncated difference contrast, and the difference contrast generally gives the lowest values among the three. Finally, in all scenarios, the values of all methods increase as the sample size increases. At the sample size of 500, the values of CWL with the win indicator are already very close to the theoretical optimal values (dashed horizontal lines in the figure).

### 4.2 Ordinal outcome

Ordinal outcomes are generated by cumulative logit models with proportional odds. Let $Y_{i},i=1\ldots ,n$ be independent and take integer values 1,…,J. Suppose $Pr(Y_{i}\leq j)=p_{ij}$ for j=1…,J−1, where $p_{ij}$ satisfies

$$\log\left(\frac{p_{ij}}{1-p_{ij}}\right)=\theta_{j}-\mu (X_{i})-A_{i}T(X_{i}),\;\text{for}\;j=1,\ldots ,J-1,$$

where $\theta_{1}<\cdots <\theta_{J-1}$ are baseline log odds ratios. As in Section 4.1, $X_{i},i=1,\ldots ,n$ consists of independent uniform random variables on [−1,1]. The same true propensity score model as in Section 4.1 is used. We fix J=4 and consider two sets of baseline coefficients: $\left(\theta_{1},\ldots ,\theta_{J-1})=(1,2,3\right)$ and (−1.5,0,1.5), which correspond to baseline category probability distributions (0.73, 0.15, 0.072, 0.047) and (0.18, 0.32, 0.32, 0.18). These two settings represent uneven and nearly even distribution of categories of the ordinal outcome, respectively. The following two scenarios are considered:

(2.a) Linear $\mu (X_{i})=X_{i3}-X_{i4}$ and linear $T(X_{i})=0.5+X_{i1}-X_{i2}$;
(2.b) Nonlinear $\mu (X_{i})=X_{i3}^{2}-X_{i4}^{2}$ and nonlinear $T(X_{i})=5(0.5-X_{i1}^{2}-X_{i2}^{2})$.

To accommodate the ordinal responses, Q‐learning is modified to be an proportional odds model with main effects and interactions between treatment and each covariate. In outcome weighted learning, the ordinal outcomes are treated as continuous values (1‐4) when used in the weights. In RWL, a proportional odds model with the main effects of all covariates is fitted. The residuals are defined as the difference between the observed value of the outcome and the weighed sum of all possible values of the outcome with the fitted probabilities as the weights. The outcome model in EARL is a linear model on the value of the outcome. We again consider sample sizes 50, 150, and 500, and generate a validation dataset of size 50 000 in each simulation replication to calculate the values of all methods as in Section 4.1 treating the ordinal outcome as a continuous variable.

**FIGURE 2:**
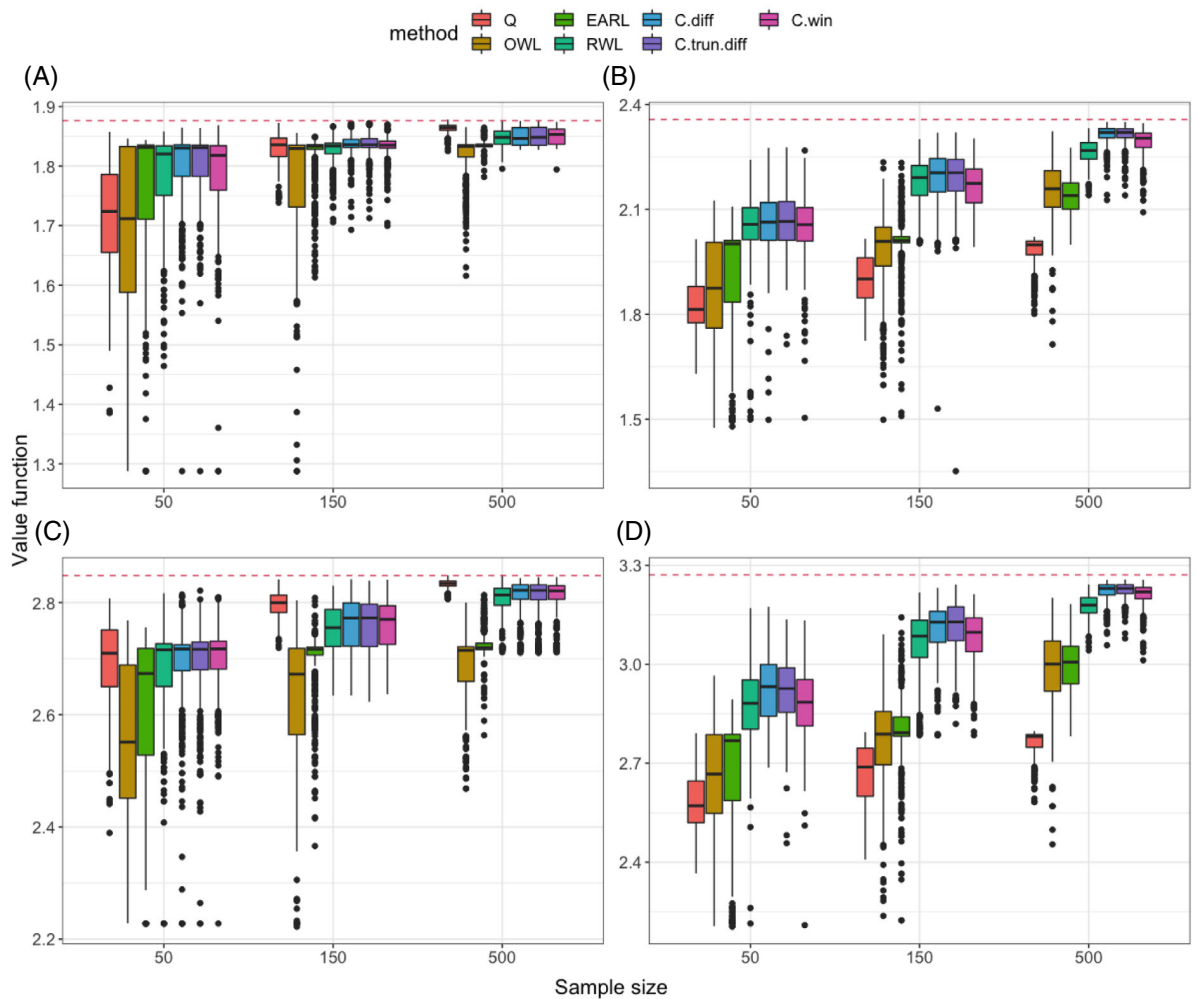
Testing value functions in 400 replications for 4 scenarios with ordinal outcomes. The seven methods we compare (from left to right in plots) are: Q, OWL, EARL, RWL, and CWL with three types of contrasts (C.diff, C.trun.diff, C.win). The dashed horizontal line represents the theoretical optimal value. (A) Linear, uneven; (B) nonlinear, uneven; (C) linear, even; (D) nonlinear, even

The simulation results for ordinal outcomes (Figure 2) also illustrate the advantages of CWL over existing methods. The advantages are again more obvious in the scenarios with nonlinear boundaries compared to those with linear boundaries. A new observation that is different from the continuous outcome scenarios is that CWL with difference contrast works slightly better than the other two contrasts under nonlinear boundary. This may be explained by the fact that the ordinal outcome is bounded without outlier or heavy tail so under this conventional condition the difference contrast makes better use of information in the ordering of categories of the outcome. Another observation is that the relative performance of Q‐learning, EARL, and RWL compared to CWL on ordinal outcomes is worse than that on continuous outcomes under the conventional setting (1.a). This may be due to the fact that modeling ordinal outcome is more challenging than fitting linear regression model. On the other hand, CWL, as a model‐free method, avoids the bias caused by potential model misspecification.

### 4.3 Additional simulations

**FIGURE 3:**
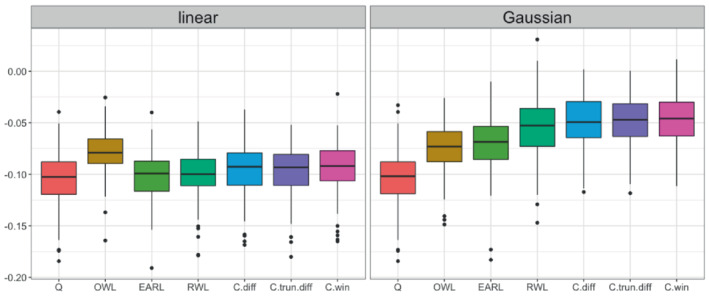
Testing value functions in 100 replicated cross‐validations for the ACE‐IPF trial data with continuous outcomes

In this section, we conduct additional simulations with more scenarios and methods. We simulate continuous outcomes with outliers with a smaller magnitude of $\mu_{O}=10$ and generate less skewed continuous outcomes with $\sigma_{\log}=1$. Under these settings, CWL with win indicator and truncated difference contrasts are still the top performers although their margin over the other methods decreases, largely due to the improved values in the other methods. We also conduct simulations to check the impact of a misspecified propensity score model on OTR estimation. The results (see Figure 3 in Web Appendix 2.2) suggests that our proposed methods are not sensitive to the misspecification of propensity score model in our simulations. Moreover, we explore the effect of unrelated covariates on OTR estimation. In particular, we increase the dimension of covariates from 5 to 10 by introducing 5 additional unrelated covariates under the nonlinear boundary scenarios with continuous outcomes. The value of all methods declines after introducing the unrelated covariates, which is not surprising given that the same sample sizes are used when p=5 and p=10. Nevertheless, CWL methods still outperform the existing methods, and the margins increases with sample size. The results of the above additional simulations are included in Web Appendix 2.2 of online Supporting Information. The numerical summary of the results in Figures 1 and 2 can also be found in Web Appendix 2.2. In addition to the values of the methods under investigation, we also record their computation time. The median computation time of one replication in scenarios (1.a) and (1.b) for all methods is reported in Table C1 in Appendix C. The computation efficiency of CWL methods is very reasonable and comparable to that of Q‐learning and OWL. In contrast, the computation time of RWL and EARL is substantially longer, especially with nonlinear kernels.

## 5 REAL DATA ANALYSIS

We apply the proposed CWL method to two real clinical datasets from the trials ACE‐IPF^12^ and ORCHID^14^ with continuous and ordinal outcomes, respectively.

### 5.1 ACE‐IPF trial with continuous outcome

ACE‐IPF trial aimed to demonstrate the efficacy of 48‐week warfarin treatment on IPF patients. The study randomized 145 patients, in a 1:1 ratio, to either warfarin or placebo group. One endpoint of interest is the percent change of lung FVC from baseline to week 48. We use 111 patients (54 in treatment group, 57 in placebo group) with a non‐missing endpoint in our analysis. The distribution of the percent change of FVC shows slight skewness to the left (skewness^25^ = −0.56 ) with one possible outlier. The histogram of the outcome and detailed skewness calculation are included in Web Appendix 2.3.

The characteristics of patients used to recommend treatment assignments include four demographics (age, gender, race, smoke status) and one clinical variable (total lung capacity) collected at enrollment. Continuous covariates are standardized before estimating OTR. Even though the true propensity score is known by the randomization design, we still estimate it by an $L^{1}$‐penalized logistic regression as it is shown that using the estimated instead of the true propensity score leads to a more efficient estimate of the average treatment effect.^26^ Moreover, the 23% missingness in the endpoint may break the balance in covariates achieved by the original randomization. In Q‐learning and EARL, we fit a linear model with the main effect of treatment and the above covariates and their pairwise interactions. In RWL, the main effect model includes all covariates. For all methods except Q‐learning, we use both linear and Gaussian kernels to estimate the OTR.

We analyze the data with cross‐validation to make the values comparable across methods and to prevent overfitting. The full dataset is randomly partitioned into four equal‐sized folds. We estimate the OTR based on three folds and calculate the value of the estimated OTR using the remaining fold. This step is repeated over the four folds to generate four values, the average of which is the cross‐validation value of the OTR. The validating value function for each validating fold of size m is given by $\left[m^{-1}\sum_{i=1}^{m}Y_{i}\cdot I(A_{i}=\hat{d}(X_{i}))/\hat{\pi }(A_{i},X_{i})]/[m^{-1}\sum_{i=1}^{m}I(A_{i}=\hat{d}(X_{i}))/\hat{\pi }(A_{i},X_{i})\right]$, where $\hat{d}$ is the rule estimated using the training folds and $\hat{\pi }$ is obtained by fitting $L^{1}$‐penalized logistic regression to the validating fold. This cross‐validation procedure is repeated 100 times and the resulting values are summarized by boxplots in Figure 3. Since an increase in FVC is desirable, methods with a higher value are considered to have better performance. The numerical summary of the values of all methods is included in Web Appendix 2.3 of online Supplementary Materials.

With a linear kernel, all methods have similar values except for the OWL, which gives a higher value. With a Gaussian kernel, the values of all classification‐based methods increase substantially with the three CWL methods generating the highest values. This result suggests that a nonlinear treatment decision rule offers a much better outcome than a linear treatment rule for this dataset, and CWL methods are advantageous over existing OTR estimation methods even with mild skewness and outlier in the outcome. To gain some insights into the estimated OTR, we also implement each method with the full dataset and summarize the subject characteristics of the control and treatment groups based on the estimated rules. Taking the CWL with the win indicator contrast under Gaussian kernel (the best method in Figure 3) as an example, it assigns warfarin treatment to 45% of subjects which is less than the observed treatment percentage of 48.6%. It tends to assign warfarin treatment to younger female subjects who never smoke and with lower baseline total lung capacity. A complete summary of subject characteristics by recommended group for all methods under Gaussian kernel is included in the Web Table 4.

### 5.2 ORCHID trial with ordinal outcome

ORCHID trial aimed to determine whether hydroxychloroquine is an efficacious treatment for adults hospitalized with COVID‐19. In this trial, 479 inpatients are randomized 1:1 to either hydroxychloroquine or placebo group and received 10 doses of the assigned treatment over 5 days. The primary outcome was the World Health Organization recommended COVID outcomes scale 14 days after randomization.^15^ It is a 7‐category ordinal scale ranging from 1 (death) to 7 (discharged from the hospital and able to perform normal activities). The original analysis did not find a significant difference in the primary outcome between the two study groups.^14^ We seek to further identify an optimal treatment decision rule for hydroxychloroquine that could potentially benefit patients with certain baseline characteristics. Four hundred and forty‐five subjects who completed the day 15 assessment are included in our analysis including 219 in treatment group and 226 in control group. The covariates for determining the decision rule are age, BMI, gender (male/female), race (white/black/other), baseline Sequential Organ Failure Assessment score, and baseline ICU admission. Again, all continuous covariates are standardized before estimating the OTR. All methods are implemented as described in Section 4.2.

**FIGURE 4:**
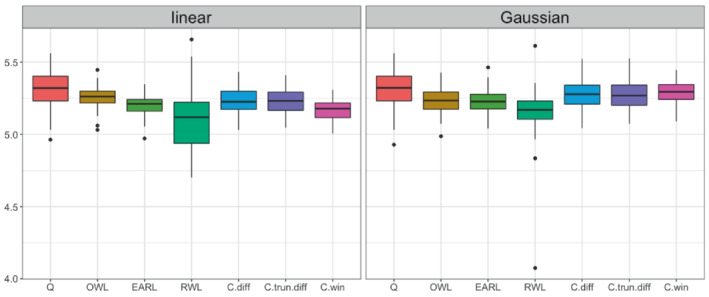
Testing value functions in 100 replicated cross‐validations for the ORCHID trial data with ordinal outcomes

Figure 4 summarizes the values of the seven methods. Since higher categories of the COVID outcome scale represents a better clinical condition, methods that generate higher values are considered to have better performance. For this dataset, the choice of linear or nonlinear decision functions does not make a discernible difference for the existing OTR methods, but a nonlinear decision function with a Gaussian kernel improves the values of CWL methods, especially the one with win indicator contrast. However, the values of CWL methods are slightly lower than that of the Q‐learning method. The reason that we do not observe a significant advantage of CWL over existing methods as found in the simulation studies may be that the overall effect size of hydroxychloroquine is very small. The median outcome scale is 6 in both hydroxychloroquine and placebo group and the adjusted odds ratio between the two groups is only 1.02 (95% CI 0.73 to 1.42).^14^

## 6 DISCUSSION

In this article, we propose the contrast weighted learning (CWL), a novel optimal treatment rule estimation method that is based on contrast functions between outcomes. Although we have focused on demonstrating the robustness of CWL to ill‐distributed scalar outcome variables, it is also a versatile OTR learning tool that can potentially accommodate a broad range of outcomes including censored, multivariate, high dimensional (eg, gene expression and medical images), or functional data as long as a scalar contrast function that satisfies conditions (i) to (iv) can be defined between two subjects. In many situations, the scalar pairwise contrast is much easier to work with for OTR estimation than the original outcome, especially for complex outcomes.

A critical component of the CWL methods is the choice of the contrast function. An overly robust contrast function may make CWL inferior to existing OTR methods due to its inefficient use of information. In practice, the choice of contrast should be driven by the distributional characteristics of the outcome. Robust contrasts such as win indicator generally work better for ill‐distributed outcomes whereas less robust contrasts such as difference contrast may be preferred for well‐behaved or bounded outcomes. Practitioners are encouraged to conduct exploratory data analysis on the outcome distribution to guide their *a priori* decisions on the contrast function. Alternatively, practitioners can use the repeated cross‐validation approach in the real data analysis section as an *ad‐hoc* method to identify the optimal contrast function from several candidates with different levels of robustness. The candidates can also include existing OTR methods. One useful strategy is to consider a family of candidate contrast functions indexed by certain parameters. An example of such a family would be the truncated difference contrasts indexed by the truncation cutoff t.

Large‐scale clinical data are increasingly available due to the advance in modern data capturing technology. To leverage the rich clinical data to generate a useful treatment decision rule, researchers tend to include a large number of covariates when estimating the OTR. Thus, the computational efficiency of an OTR learning method becomes an important consideration for its practical utility. In the simulation studies, we use the best implementation of the OTR methods that we are aware of. In addition to its great flexibility and robustness, the proposed CWL also offers attractive computational performance under both linear and Gaussian kernels as shown in Table C1 in Appendix. In general, the regression‐based (Q‐learning) and SVM‐based methods (OWL, CWL) have an obvious advantage in computational efficiency than RWL and EARL. The difference is dramatically magnified under the Gaussian kernel, likely due to the optimization algorithms and more tuning parameters in RWL and EARL. The excessive computation time of RWL and EARL hinders sufficient tuning of their parameters and thus may affect their performance in practice. As shown in our real data analysis of ACE‐IPF trial, the Gaussian kernel can identify a much better OTR over the linear kernel in practice. Therefore, it is particularly advantageous for the CWL to offer superior computation performance under the Gaussian kernel.

Like many weighted‐learning methods for OTR estimation, one limitation of CWL is that its consistency depends on the correct specification of the treatment model. The EARL method possesses the doubly robust property where its consistency only requires the correct specification of either the treatment model or the outcome model.^8^ In practice, however, the specification of neither model is testable, and it is likely that both models are misspecified. There is some evidence that a misspecified outcome model has a more detrimental effect on OTR performance than a misspecified treatment model.^8^ Our simulation study also demonstrates that the finite sample performance of weighted‐learning methods including CWL is not sensitive to treatment model misspecification. Therefore, we argue that it is generally preferable to model treatment instead of outcome as do the OWL and CWL. Wu et al^27^ recently proposed a matched OTR learning method that avoids fitting a treatment model by nonparametrically matching patients based on their characteristics. It would be an interesting future research direction to incorporate matching to CWL to make it even more robust.

## Supporting information


**Data S1**: Supporting Information

## Data Availability

The data used as the real data analysis examples are openly available in BioLINCC at https://biolincc.nhlbi.nih.gov/home/, accession number HLB01131414a and HLB02372021a.

## APPENDIX A DERIVATION OF RISK FUNCTION OF CWL

### A.1

We first prove that $f^{*}={\text{arg max}}_{f}V(f)$ is the minimizer of the risk function (3). Maximizing value function V(f) with respect to f is equivalent to minimizing the risk function

$$\tilde{R}(f)=\frac{1}{2}E\left[\frac{h(Y_{i},Y_{j})}{\pi (A_{i},X_{i})\pi (A_{j},X_{j})}\right]-V(f)=\frac{1}{2}E\left\{[I(A_{i}f(X_{i})<0)+I(A_{j}f(X_{j})\geq 0)]\frac{h(Y_{i},Y_{j})}{\pi (A_{i},X_{i})\pi (A_{j},X_{j})}\right\},$$

where the second equality uses the condition h(x,y)=−h(y,x). From the perspective of weighted classification, $\tilde{R}(f)$ defines a joint loss $I(A_{i}f(X_{i})<0)+I(A_{j}f(X_{j})\geq 0)$ for a given pair (i,j), and assigns a contrast‐based weight $h(Y_{i},Y_{j})/[\pi (A_{i},X_{i})\pi (A_{j},X_{j})]$ to it. Since the weight can be negative, we need to separate its scale and sign as follows:

$$\begin{array}{llll} \tilde{R}(f) & =\frac{1}{2}E\{[I(\text{sgn}(h(Y_{i},Y_{j}))A_{i}f(X_{i})<0)+I(\text{sgn}(h(Y_{i},Y_{j}))A_{j}f(X_{j}))\geq 0)]\frac{\left|h(Y_{i},Y_{j})\right|}{\pi (A_{i},X_{i})\pi (A_{j},X_{j})}\}\; & & \\ & \;-\frac{1}{2}E\left[\frac{\left|h(Y_{i},Y_{j})\right|}{\pi (A_{i},X_{i})\pi (A_{j},X_{j})}\right][1-\text{sgn}(h(Y_{i},Y_{j}))].\; & & \end{array}$$

The term in the last line does not involve f, we therefore omit it in the optimization to get the risk function R(f) in (3) and the optimal decision function $f^{*}={\text{arg min}}_{f}\tilde{R}(f)={\text{arg min}}_{f}R(f)$.

Now, we derive the transformed empirical risk function in (5). Without loss of generality, assume the outcome in the data has been sorted in descending order, that is, $Y_{1}\geq \cdots \geq Y_{n}$. Then, for any i<j, $\text{sgn}(h(Y_{i},Y_{j}))\geq 0$ by condition (ii) of h, and the empirical risk can be estimated as

R^ϕ(f)=12n2−1∑i<j{[ϕ(Aif(Xi))+ϕ(−Ajf(Xj))]h(Yi,Yj)π(Ai,Xi)π(Aj,Xj)},

where ϕ(x)=max(0,1−x) is the hinge loss function. Let $c_{ij}=h(Y_{i},Y_{j})/\pi (A_{j},X_{j})$ for any i,j and note that $c_{ij}=0$ if i=j,

R^ϕ(f)=12n2−1[∑i=1n∑j=incijϕ(Aif(Xi))π(Ai,Xi)+∑j=1n∑i=1jcjiϕ(−Ajf(Xj))π(Aj,Xj)]=12n2−1∑k=1n[ϕ(Akf(Xk))(∑j=knckj)+ϕ(−Akf(Xk))(∑i=1kcik)]/π(Ak,Xk).

To minimize the empirical risk function, we only need to consider −1≤f(X)≤1 since outside of [−1,1] the objective function takes greater or equal values than those at 1 or −1. Rewrite the risk function after removing the terms not depending on f,

R^ϕ(f)=12n2−1∑k=1n[−Akf(Xk)(∑j=knckj)+Akf(Xk)(∑i=1kcik)]/π(Ak,Xk)=12n2−1∑k=1n−Akf(Xk)(∑i=k+1ncki−∑i=1k−1cik)/π(Ak,Xk)=14n∑k=1n−Akf(Xk)Ck,nhπ(Ak,Xk),

where $C_{k,n}^{h}={\left(n-1\right)}^{-1}\sum_{i\neq k}h(Y_{k},Y_{i})/\pi (A_{i},X_{i})$, given contrast h, for any k=1,...,n. Rewrite

$${\hat{R}}_{\phi }(f)=\frac{1}{4n}\overset{n}{\underset{k=1}{\sum }}-\text{sgn}(C_{k,n}^{h})A_{k}f(X_{k})\frac{\left|C_{k,n}^{h}\right|}{\pi (A_{k},X_{k})},$$

Equivalently, we can minimize the following empirical risk function:

$${\hat{R}}_{\phi }(f)=\frac{1}{n}\overset{n}{\underset{i=1}{\sum }}\phi ({Ã}_{i}f(X_{i}))\frac{\left|C_{i,n}^{h}\right|}{\pi (A_{i},X_{i})},$$

where ${Ã}_{i}=\text{sgn}(C_{i,n}^{h})A_{i}$ is a pseudo treatment assignment, which takes on the opposite value of the observed assignment if and only if the average contrast between subject i and all other subjects is below zero.

## APPENDIX B PROOF OF THEOREM 1 (FISHER CONSISTENCY)

### B.1

Given the risk function R(d), the optimal treatment rule $d^{*}(x)$ is the Bayes decision rule, which equals $\text{sgn}\{E[h(Y^{\left(1\right)},Y^{\left(-1\right)})|X=x]\}$ for all x, where $Y^{\left(a\right)}=$ is the potential outcome for treatment assignment a∈{−1,1}. By condition (iii) of the contrast function h, h(x,y)=−h(y,x), the ϕ‐risk function has:

$$\begin{array}{llll} R_{\phi }(f) & =\frac{1}{2}E\{\frac{\left[\phi (\text{sgn}(h(Y_{i},Y_{j}))A_{i}f(X_{i}))+\phi (-\text{sgn}(h(Y_{i},Y_{j}))A_{j}f(X_{j}))]\cdot |h(Y_{i},Y_{j})\right|}{\pi (A_{i},X_{i})\pi (A_{j},X_{j})}\}\; & & \\ & =\frac{1}{2}E\{\phi (\text{sgn}(h(Y_{i},Y_{j}))A_{i}f(X_{i}))\cdot \frac{\left|h(Y_{i},Y_{j})\right|}{\pi (A_{i},X_{i})\pi (A_{j},X_{j})}+\phi (\text{sgn}(h(Y_{j},Y_{i}))A_{j}f(X_{j}))\cdot \frac{\left|h(Y_{j},Y_{i})\right|}{\pi (A_{j},X_{j})\pi (A_{i},X_{i})}\}\; & & \\ & =E\{\phi (\text{sgn}(h(Y_{i},Y_{j}))A_{i}f(X_{i}))\cdot \frac{\left|h(Y_{i},Y_{j})\right|}{\pi (A_{i},X_{i})\pi (A_{j},X_{j})}\}.\; & & \end{array}$$

The last equality is due to i.i.d. $\left(Y_{i},A_{i},X_{i}\right)$. Given $X_{i}=x_{i},X_{j}=x_{j}$, consider $-1\leq f(x_{i}),f(x_{j})\leq 1$,

$$\begin{array}{llll} & E\{[\phi (\text{sgn}(h(Y_{i},Y_{j}))\cdot A_{i}f(X_{i}))]\frac{\left|h(Y_{i},Y_{j})\right|}{\pi (A_{i},X_{i})\pi (A_{j},X_{j})}|X_{i}=x_{i},X_{j}=x_{j}\}\; & & \\ & \;=E\{\frac{-f(x_{i})h(Y_{i},Y_{j})}{\pi (1,x_{i})\pi (1,x_{j})}|A_{i}=1,A_{j}=1,X_{i}=x_{i},X_{j}=x_{j}\}\pi (1,x_{i})\pi (1,x_{j})\; & & \\ & \;+E\{\frac{f(x_{i})h(Y_{i},Y_{j})}{\pi (1,x_{i})\pi (-1,x_{j})}|A_{i}=1,A_{j}=-1,X_{i}=x_{i},X_{j}=x_{j}\}\pi (1,x_{i})\pi (-1,x_{j})\; & & \\ & \;+E\{\frac{-f(x_{i})h(Y_{i},Y_{j})}{\pi (-1,x_{i})\pi (1,x_{j})}|A_{i}=-1,A_{j}=1,X_{i}=x_{i},X_{j}=x_{j}\}\pi (-1,x_{i})\pi (1,x_{j})\; & & \\ & \;+E\{\frac{f(x_{i})h(Y_{i},Y_{j})}{\pi (-1,x_{i})\pi (-1,x_{j})}|A_{i}=-1,A_{j}=-1,X_{i}=x_{i},X_{j}=x_{j}\}\pi (-1,x_{i})\pi (-1,x_{j})+c\; & & \\ & \;=f(x_{i})E[h(Y_{i}^{\left(-1\right)},Y_{j}^{\left(1\right)})-h(Y_{i}^{\left(1\right)},Y_{j}^{\left(1\right)})+h(Y_{i}^{\left(-1\right)},Y_{j}^{\left(-1\right)})-h(Y_{i}^{\left(1\right)},Y_{j}^{\left(-1\right)})|X_{i}=x_{i},X_{j}=x_{j}]+c,\; & & \end{array}$$

where c represent other terms unrelated to f. As the minimizer of $R_{\phi }(f)$, $\tilde{f}$ satisfies that $\text{sgn}(\tilde{f})=\text{sgn}\{E[h(Y_{i}^{\left(1\right)},Y_{j}^{\left(1\right)})-h(Y_{i}^{\left(-1\right)},Y_{j}^{\left(1\right)})+h(Y_{i}^{\left(1\right)},Y_{j}^{\left(-1\right)})-h(Y_{i}^{\left(-1\right)},Y_{j}^{\left(-1\right)})|X_{i}=x_{i},X_{j}=x_{j}]\}$. By the conditions (i)‐(iv) of h, we have $h(x_{1},y)>h(x_{2},y)$ if and only if $h(x_{1},x_{2})>0$ for any y. Then, $\text{sgn}(\tilde{f})=\text{sgn}\{E[h(Y_{i}^{\left(1\right)},Y_{i}^{\left(-1\right)})|X_{i}=x_{i}]\}$, which is the Bayes rule $d^{*}$.

## APPENDIX C COMPUTATIONAL PERFORMANCE

### C.1

**TABLE C1:** Median computing time (seconds) of one run in 400 replications of scenarios (1.a) and (1.b), using linear and Gaussian kernels respectively

Kernel	n	Q	OWL	C.diff	C.trun.diff	C.win	RWL	EARL
Linear	50	0.22	0.58	0.30	0.31	0.28	2.82	3.82
	150	0.21	1.25	0.70	0.69	0.48	3.98	3.78
	500	0.21	6.16	3.13	2.92	1.43	7.37	4.57
Gaussian	50	0.37	0.29	0.24	0.24	0.24	25.32	112.98
	150	0.20	0.45	0.35	0.35	0.35	246.19	751.83
	500	0.21	1.54	0.89	0.91	0.91	1934.01	14915.73

*Note*: The numbers of candidate tuning parameters for all the method (not applicable to Q‐learning) are identical.
