# Supplementary material for: Contrast weighted learning for robust optimal treatment rule estimation
Source: Stat Med. 2022 Sep 14;41(27):5379–94. doi: 10.1002/sim.9574 (PMC9826186; doi:10.1002/sim.9574)
Supplement: Supplementary file 1 — Data S1: Supporting Information [file SIM-41-5379-s001.pdf]

Received: Added at production | Revised: Added at production | Accepted: Added at production

DOI: xxx/xxxx

RESEARCH ARTICLE

Supporting Information for "Contrast Weighted Learning for Robust Optimal Treatment Regimens Estimation" by Xiaohan Guo and Ai Ni

Xiaohan Guo | Ai Ni*

[1]Division of Biostatistics, College of Public Health, The Ohio State University, Ohio, U.S.

Correspondence
Ai Ni, 1841 Neil Ave. 280D Cunz Hall Columbus, OH 43210. Email: ni.304@osu.edu

Abstract

Web Appendix 1 includes additional theoretical results and detailed proof. We derive the excess risk for the proposed contrast weighted learning (CWL) in Web Appendix 1.1. Then, the proof of convergence rate of the estimated rule via CWL in Theorem 2 of the main article is presented in Web Appendix 1.2. Web Appendix 2.1 contains the details on the optimization of a weighted SVM problem. Web Appendix 2.2 includes the additional simulation results for the parameter settings that are different from the ones in the main article. Web Appendix 2.3 presents the exploratory data analysis for the two real datasets we used in the main article.

KEYWORDS:
Contrast function; Individualized treatment rule; Ordinal outcome; Outcome weighted learning; Personalized medicine; Robustness.

Web Appendix 1 | THEORETICAL RESULTS AND PROOFS

Web Appendix 1.1 | Excess risk

To derive the convergence rate of empirical risk function $\hat{R}(f)$, we first need to validate that the excess risk of decision function f under 0-1 loss is bounded by the excess risk of f under hinge loss ϕ since the estimated optimal treatment rule (OTR) is the minimizer of a (penalized) ϕ-risk function. Considering i.i.d. triplet $(Y_i, A_i, \mathbf{X}_i)$ for $i = 1, \dots, n$, $Y_i \in \mathbb{R}$, $A_i \in \{-1, 1\}$, and $\mathbf{X}_i \in \mathcal{X} \subset \mathbb{R}^p$. Recall that the risk function $R(f)$ is defined as

$$R(f) = \frac{1}{2} E\left[(I(A_i f(\mathbf{X}_i) < 0) + I(A_j f(\mathbf{X}_j) \geq 0)) \frac{h(Y_i, Y_j)}{\pi(A_i, \mathbf{X}_i)\pi(A_j, \mathbf{X}_j)} \right] = E\left[\frac{I(A_i f(\mathbf{X}_i) < 0) h(Y_i, Y_j)}{\pi(A_i, \mathbf{X}_i)\pi(A_j, \mathbf{X}_j)} \right],$$

[0]**Abbreviations:** OTR, optimal treatment rule; CWL, contrast weighted learning; OWL, outcome weighted learning; RWL, residual weighted learning; EARL,

where $\pi(A_i, \mathbf{X}_i) = P(A_i \mid \mathbf{X}_i)$ and $h(Y_i, Y_j)$ is a contrast function that satisfies conditions (i)-(iv) in the main article. The simplification above follows almost the same lines in the proof of Fisher consistency in Appendix. The corresponding ϕ-risk is

$$R_\phi(f) = E\left[\frac{\phi(\text{sgn}(h(Y_i, Y_j))A_i f(\mathbf{X}_i))|h(Y_i, Y_j)|}{\pi(A_i, \mathbf{X}_i)\pi(A_j, \mathbf{X}_j)}\right],$$

where $\phi(x) = \max(0, 1 - x)$. Note that the second term does not depends on f.

Lemma 1 (Excess risk)**.** For any measurable decision function f,

$$R(f) - R^* \leq R_\phi(f) - R_\phi^*,$$

where $R^* = \inf_f R(f)$, $R_\phi^* = \inf_f R_\phi(f)$.

Proof. Here, we only show the proof for discrete outcome Y_i and the counterpart for continuous outcome follows almost the same lines but replaces the summation with integral.

$$\begin{aligned} R(f) &= E\left\{E\left[\frac{I(A_i f(\mathbf{X}_i) < 0)h(Y_i, Y_j)}{\pi(A_i, \mathbf{X}_i)\pi(A_j, \mathbf{X}_j)} \mid \mathbf{X}_i, \mathbf{X}_j\right]\right\} \\ &= E\left\{\sum_{y_i} P(Y_i = y_i \mid \mathbf{X}_i) E\left[\frac{h(y_i, y_j)I(A_i f(\mathbf{X}_i) < 0)}{\pi(A_i, \mathbf{X}_i)\pi(A_j, \mathbf{X}_j)} \mid Y_i = y_i, \mathbf{X}_i, \mathbf{X}_j\right]\right\} \\ &= E\left\{\sum_{y_i} P(Y_i = y_i \mid \mathbf{X}_i)\left[\sum_{y_j} \frac{h(y_i, y_j)}{\pi(A_j, \mathbf{X}_j)} P(Y_j = y_j \mid \mathbf{X}_j)\right] \times E\left[\frac{I(A_i f(\mathbf{X}_i) < 0)}{\pi(A_i, \mathbf{X}_i)} \mid Y_i = y_i, \mathbf{X}_i\right]\right\} \end{aligned}$$

For notation simplicity, let

$$q_{y_i} = q_{y_i}(\mathbf{X}_i, \mathbf{X}_j) = P(Y_i = y_i \mid \mathbf{X}_i)\left[\sum_{y_j} \frac{h(y_i, y_j)}{\pi(A_j, \mathbf{X}_j)} P(Y_j = y_j \mid \mathbf{X}_j)\right],$$

then rewrite the risk function:

$$\begin{aligned} R(f) =& E\left\{\sum_{y_i} q_{y_i} E\left[\frac{I(A_i f(\mathbf{X}_i) < 0)}{\pi(A_i, \mathbf{X}_i)} \mid Y_i = y_i, \mathbf{X}_i\right]\right\} \\ =& E\left\{\sum_{y_i} |q_{y_i}| E\left[\frac{\text{sgn}(q_{y_i}) I(A_i f(\mathbf{X}_i) < 0)}{\pi(A_i, \mathbf{X}_i)} \mid Y_i = y_i, \mathbf{X}_i\right]\right\} \\ =& E\left\{\sum_{y_i} |q_{y_i}| E\left[\frac{I(\text{sgn}(q_{y_i}) A_i f(\mathbf{X}_i) < 0)}{\pi(A_i, \mathbf{X}_i)} + \frac{\text{sgn}(q_{y_i}) - 1}{2\pi(A_i, \mathbf{X}_i)} \mid Y_i = y_i, \mathbf{X}_i\right]\right\} \end{aligned}$$

Define $\eta_{y_i}(\mathbf{X}_i) = P(A_i = 1 \mid Y_i = y_i, \mathbf{X}_i)$. The risk function can be further decomposed as:

$$
\begin{aligned}
R(f) =& E\left\{ \sum_{y_i} |q_{y_i}| E\left[\frac{I(\mathrm{sgn}(q_{y_i}) A_i f(\mathbf{X}_i) < 0)}{\pi(A_i, \mathbf{X}_i)} + \frac{\mathrm{sgn}(q_{y_i}) - 1}{2\pi(A_i, \mathbf{X}_i)} \mid Y_i = y_i, \mathbf{X}_i \right] \right\} \\
=& E\left\{ \sum_{y_i} |q_{y_i}| E\left[\frac{I(\mathrm{sgn}(q_{y_i}) A_i f(\mathbf{X}_i) < 0)}{\pi(A_i, \mathbf{X}_i)} \mid A_i = 1, Y_i = y_i, \mathbf{X}_i \right] \eta_{y_i}(\mathbf{X}_i) \right. \\
& \left. + \sum_{y_i} |q_{y_i}| E\left[\frac{I(\mathrm{sgn}(q_{y_i}) A_i f(\mathbf{X}_i) < 0)}{\pi(A_i, \mathbf{X}_i)} \mid A_i = -1, Y_i = y_i, \mathbf{X}_i \right] (1 - \eta_{y_i}(\mathbf{X}_i)) \right\} \\
& + E\left\{ \sum_{y_i} |q_{y_i}| E\left[\frac{\mathrm{sgn}(q_{y_i}) - 1}{2\pi(A_i, \mathbf{X}_i)} \mid Y_i = y_i, \mathbf{X}_i \right] \right\} \\
=& E\left\{ \sum_{y_i} |q_{y_i}| \left[\frac{\eta_{y_i}(\mathbf{X}_i)}{\pi(1, \mathbf{X}_i)} I(q_{y_i} f(\mathbf{X}_i) < 0) + \frac{1 - \eta_{y_i}(\mathbf{X}_i)}{\pi(-1, \mathbf{X}_i)} I(q_{y_i} f(\mathbf{X}_i) \geq 0) \right] \right\} + E\left\{ \sum_{y_i} |q_{y_i}| E\left[\frac{\mathrm{sgn}(q_{y_i}) - 1}{2\pi(A_i, \mathbf{X}_i)} \mid Y_i = y_i, \mathbf{X}_i \right] \right\} \\
=& E\left\{ \sum_{y_i : q_{y_i} \geq 0} |q_{y_i}| \left[\frac{\eta_{y_i}(\mathbf{X}_i)}{\pi(1, \mathbf{X}_i)} I(f(\mathbf{X}_i) < 0) \right] + \sum_{y_i : q_{y_i} < 0} |q_{y_i}| \left[\frac{\eta_{y_i}(\mathbf{X}_i)}{\pi(1, \mathbf{X}_i)} I(f(\mathbf{X}_i) \geq 0) \right] + \sum_{y_i : q_{y_i} \geq 0} |q_{y_i}| \left[\frac{1 - \eta_{y_i}(\mathbf{X}_i)}{\pi(-1, \mathbf{X}_i)} I(f(\mathbf{X}_i) < 0) \right] \right. \\
& \left. + \sum_{y_i : q_{y_i} < 0} |q_{y_i}| \left[\frac{1 - \eta_{y_i}(\mathbf{X}_i)}{\pi(-1, \mathbf{X}_i)} I(f(\mathbf{X}_i) \geq 0) \right] \right\} + E\left\{ \sum_{y_i} |q_{y_i}| E\left[\frac{\mathrm{sgn}(q_{y_i}) - 1}{2\pi(A_i, \mathbf{X}_i)} \mid Y_i = y_i, \mathbf{X}_i \right] \right\} \\
=& E\left\{ z_0(\mathbf{X}_i, \mathbf{X}_j) \left[\tau(\mathbf{X}_i, \mathbf{X}_j) I(f(\mathbf{X}_i) < 0) + (1 - \tau(\mathbf{X}_i, \mathbf{X}_j)) I(f(\mathbf{X}_i) \geq 0) \right] \right\} + E\left\{ \sum_{y_i} |q_{y_i}| E\left[\frac{\mathrm{sgn}(q_{y_i}) - 1}{2\pi(A_i, \mathbf{X}_i)} \mid Y_i = y_i, \mathbf{X}_i \right] \right\},
\end{aligned}
\tag{1}
$$

where

$$
\tau(\mathbf{X}_i, \mathbf{X}_j) = \left[\sum_{y_i : q_{y_i} \geq 0} \frac{q_{y_i} \eta_{y_i}(\mathbf{X}_i)}{\pi(1, \mathbf{X}_i)} \right] \Big/ z_0(\mathbf{X}_i, \mathbf{X}_j),
$$

$$
z_0(\mathbf{X}_i, \mathbf{X}_j) = \sum_{y_i} |q_{y_i}| \left[\frac{\eta_{y_i}(\mathbf{X}_i)}{\pi(1, \mathbf{X}_i)} + \frac{1 - \eta_{y_i}(\mathbf{X}_i)}{\pi(-1, \mathbf{X}_i)} \right].
$$

Note that $\tau(\mathbf{X}_i, \mathbf{X}_j) \in [0, 1]$ and $z_0(\mathbf{X}_i, \mathbf{X}_j) > 0$. Similarly, for the ϕ-risk, we have:

$$
R_\phi(f) = E\left\{ z_0(\mathbf{X}_i, \mathbf{X}_j) \left[\tau(\mathbf{X}_i, \mathbf{X}_j) \phi(f(\mathbf{X}_i)) + (1 - \tau(\mathbf{X}_i, \mathbf{X}_j)) \phi(-f(\mathbf{X}_i)) \right] \right\}
$$

Now, consider the function $S(\tau, \alpha) = \tau\phi(\alpha) + (1 - \tau)\phi(-\alpha)$, the optimal ϕ-risk is

$$
R^*_\phi = E[z_0(\mathbf{X}_i, \mathbf{X}_j) \inf_{\alpha \in \mathbb{R}} S(\tau(\mathbf{X}_i, \mathbf{X}_j), \alpha)],
$$

and

$$
R_\phi(f) - R^*_\phi = E\{z_0(\mathbf{X}_i, \mathbf{X}_j)[S(\tau(\mathbf{X}_i, \mathbf{X}_j), f(\mathbf{X}_i)) - \inf_{\alpha \in \mathbb{R}} S(\tau(\mathbf{X}_i, \mathbf{X}_j), \alpha)]\}.
$$

Using the results for the convexified transform of hinge loss [1], we have

$2\tau - 1 = \inf_{\alpha:\alpha(2\tau-1)\le 0} S(\tau,\alpha) - \inf_{\alpha\in\mathbb{R}} S(\tau,\alpha)$), and then according to (1),

$$\begin{aligned}
R(f) - R^* &\le E\{I(f(\mathbf{X}_i)z_0(\mathbf{X}_i,\mathbf{X}_j)[\tau(\mathbf{X}_i,\mathbf{X}_j) - 1/2] < 0)|z_0(\mathbf{X}_i,\mathbf{X}_j)[2\tau(\mathbf{X}_i,\mathbf{X}_j) - 1]|\} \\
&= E\left\{ z_0(\mathbf{X}_i,\mathbf{X}_j) I(f(\mathbf{X}_i)[\tau(\mathbf{X}_i,\mathbf{X}_j) - 1/2] < 0) \left[\inf_{\alpha:\alpha(2\tau(\mathbf{X}_i,\mathbf{X}_j)-1)\le 0} S(\tau(\mathbf{X}_i,\mathbf{X}_j),\alpha) - \inf_{\alpha\in\mathbb{R}} S(\tau(\mathbf{X}_i,\mathbf{X}_j),\alpha) \right] \right\} \\
&\le E\left\{ z_0(\mathbf{X}_i,\mathbf{X}_j) \left[S(\tau(\mathbf{X}_i,\mathbf{X}_j),\alpha) - \inf_{\alpha\in\mathbb{R}} S(\tau(\mathbf{X}_i,\mathbf{X}_j),\alpha) \right] \right\} \\
&= R_\phi(f) - R_\phi^*,
\end{aligned}$$

where the last inequality relies on the fact that, when $I(f(\mathbf{X}_i)[\tau(\mathbf{X}_i,\mathbf{X}_j) - 1/2] < 0) = 1$,

$$S(\tau(\mathbf{X}_i,\mathbf{X}_j), f(\mathbf{X}_i)) \ge \inf_{\alpha:\alpha(2\tau(\mathbf{X}_i,\mathbf{X}_j)-1)\le 0} S(\tau(\mathbf{X}_i,\mathbf{X}_j),\alpha).$$

□

Web Appendix 1.2 | Convergence rate of estimated rule

Given observed data $(Y_i, A_i, \mathbf{X}_i)$, $i = 1, \dots, n$, we estimate a decision function $\hat{f}_n$ in the reproducing kernel Hilbert space (RKHS) associated with Gaussian kernel and the estimator is given by solving the following minimization problem:

$$\hat{f}_n = \arg\min_{f\in\mathcal{H}_{\sigma_n}} \frac{1}{n} \sum_{i=1}^n \phi(\widetilde{A}_i f(\mathbf{X}_i)) \cdot \frac{|C_{i,n}^h|}{\pi(A_i,\mathbf{X}_i)} + \lambda_n \|f\|_k^2, \tag{2}$$

where $\mathcal{H}_{\sigma_n}$ is the RKHS generated by Gaussian kernel $k(\mathbf{x},\mathbf{y}) = \exp(-\sigma_n^2\|\mathbf{x}-\mathbf{y}\|^2)$, for $\mathbf{x},\mathbf{y} \in \mathbb{R}^p$, $\sigma_n > 0$; $\|\cdot\|_k$ is the norm induced by the Gaussian kernel $k(\cdot,\cdot)$. $C_{i,n}^h = (n-1)^{-1}\sum_{j\ne i} h(Y_i,Y_j)/\pi(A_j,\mathbf{X}_j)$ is the contrast weight; $\widetilde{A}_i = A_i \mathrm{sgn}(C_{i,n}^h)$ is a pseudo treatment assignment depending on the sign of $C_{i,n}^h$; $\lambda_n > 0$ is a tuning parameter.

Now, we want to investigate the convergence rate of $R(\hat{f}_n) - R^*$. By Lemma 1, it suffices to explore the convergence rate of $R_\phi(\hat{f}_n) - R_\phi^*$. Following the similar idea in many previous studies, we separate the excess ϕ-risk $R_\phi(\hat{f}_n) - R_\phi^*$ into two pieces:

$$\begin{aligned}
R_\phi(\hat{f}_n) - R_\phi^* &\le R_\phi(\hat{f}_n) + \lambda_n\|\hat{f}_n\|_k^2 - R_\phi^* \\
&= R_\phi(\hat{f}_n) + \lambda_n\|\hat{f}_n\|_k^2 - \inf_{f\in\mathcal{H}_{\sigma_n}}\{R_\phi(f) + \lambda_n\|f\|_k^2\} \qquad (3) \\
&\quad + \inf_{f\in\mathcal{H}_{\sigma_n}}\{R_\phi(f) + \lambda_n\|f\|_k^2\} - R_\phi^*, \qquad (4)
\end{aligned}$$

where (3) is the stochastic error caused by finite samples and (4) is the approximation error due to using RKHS $\mathcal{H}_{\sigma_n}$. The convergence rates for stochastic error and approximation error are derived separately.

Following the idea of Steinwart and Scovel[2], we first derive the convergence rate of approximation error. Define the best approximation function of $f \in L^2 : \mathbb{R}^p \mapsto \mathbb{R}$ in $\mathcal{H}_{\sigma_n}$ as:

$$V_{\sigma_n}(f(\mathbf{x})) = \frac{(2\sigma_n)^{p/2}}{\pi^{p/4}} \int f(\mathbf{y}) \exp(-2\sigma_n^2\|\mathbf{x}-\mathbf{y}\|^2) d\mathbf{y},$$

where $V_{\sigma_n}: L^2(\mathbb{R}^p) \mapsto \mathcal{H}_{\sigma_n}(\mathbb{R})$ is an isometric isomorphism, then

$$\begin{aligned}
&\inf_{f\in\mathcal{H}_{\sigma_n}} (R_\phi(f) + \lambda_n\|f\|_k^2 - R_\phi^*) = \inf_{g\in L^2}(R_\phi(V_{\sigma_n}(g)) + \lambda_n\|g\|^2 - R_\phi^*) \\
\leq &\inf_{g\in L^2}(R_\phi(V_{\sigma_n}(g)) - R_\phi^*) + c_p\lambda_n\sigma_n^p = R_\phi(\dot{f}) - R_\phi^* + c_p\lambda_n\sigma_n^p, \qquad (5)
\end{aligned}$$

where $\dot{f} = V_{\sigma_n}(\dot{g})$ and $\dot{g} = \arg\min_{g\in L^2} R_\phi(V_{\sigma_n}(g))$; c_p is a constant depending on p. The inequality above holds since $\|g\| \leq c_p^{1/2}\sigma_n^{p/2}$ by Steinwart and Scovel[2]. Given a contrast function $h(\cdot,\cdot)$, let h_{ij} denotes $h(Y_i, Y_j)$ for simplification of notation. Let $h_{ij}^+ = \max(h_{ij}, 0)$ and $h_{ij}^- = \max(-h_{ij}, 0)$, then $|h_{ij}| = h_{ij}^+ + h_{ij}^-$ and $h_{ij} = h_{ij}^+ - h_{ij}^-$. The ϕ-risk at $f = \dot{f}$ is:

$$\begin{aligned}
R_\phi(\dot{f}) &= E\left[\frac{\phi(\text{sgn}(h_{ij})A_i\dot{f}(\mathbf{X}_i))|h_{ij}|}{\pi(A_i,\mathbf{X}_i)\pi(A_j,\mathbf{X}_j)}\right] \\
&= \int E\left[\frac{\phi(\text{sgn}(h_{ij})A_i\dot{f}(\mathbf{X}_i))|h_{ij}|}{\pi(A_i,\mathbf{X}_i)\pi(A_j,\mathbf{X}_j)} \mid A_i = 1, \mathbf{X}_i\right]\pi(1,\mathbf{X}_i)dP(\mathbf{X}_i) \\
&\quad + \int E\left[\frac{\phi(\text{sgn}(h_{ij})A_i\dot{f}(\mathbf{X}_i))|h_{ij}|}{\pi(A_i,\mathbf{X}_i)\pi(A_j,\mathbf{X}_j)} \mid A_i = -1, \mathbf{X}_i\right]\pi(-1,\mathbf{X}_i)dP(\mathbf{X}_i) \\
&= \int E\left[\frac{\phi(\text{sgn}(h_{ij})\dot{f}(\mathbf{X}_i))(h_{ij}^+ + h_{ij}^-)}{\pi(A_j,\mathbf{X}_j)} \mid A_i = 1, \mathbf{X}_i\right]dP(\mathbf{X}_i) \\
&\quad + \int E\left[\frac{\phi(-\text{sgn}(h_{ij})\dot{f}(\mathbf{X}_i))(h_{ij}^+ + h_{ij}^-)}{\pi(A_j,\mathbf{X}_j)} \mid A_i = -1, \mathbf{X}_i\right]dP(\mathbf{X}_i) \\
&= \int \left\{E\left[\frac{h_{ij}^+}{\pi(A_j,\mathbf{X}_j)} \mid A_i = 1, \mathbf{X}_i\right] + E\left[\frac{h_{ij}^-}{\pi(A_j,\mathbf{X}_j)} \mid A_i = -1, \mathbf{X}_i\right]\right\}\phi(\dot{f}(\mathbf{X}_i))dP(\mathbf{X}_i) \\
&\quad + \int \left\{E\left[\frac{h_{ij}^+}{\pi(A_j,\mathbf{X}_j)} \mid A_i = -1, \mathbf{X}_i\right] + E\left[\frac{h_{ij}^-}{\pi(A_j,\mathbf{X}_j)} \mid A_i = 1, \mathbf{X}_i\right]\right\}\phi(-\dot{f}(\mathbf{X}_i))dP(\mathbf{X}_i) \\
&= \int E_{T_i|\mathbf{X}_i}[\phi(T_i\dot{f}(\mathbf{X}_i))]\cdot Z(\mathbf{X}_i)dP(\mathbf{X}_i),
\end{aligned}$$

where T_i takes value 1 and -1 with $P(T_i = 1 \mid \mathbf{X}_i) = \tilde{p}(\mathbf{X}_i)$,

$$\tilde{p}(\mathbf{X}_i) = \left\{E\left[\frac{h_{ij}^+}{\pi(A_j,\mathbf{X}_j)} \mid A_i = 1, \mathbf{X}_i\right] + E\left[\frac{h_{ij}^-}{\pi(A_j,\mathbf{X}_j)} \mid A_i = -1, \mathbf{X}_i\right]\right\}\Big/ Z(\mathbf{X}_i),$$

$$\begin{aligned}
Z(\mathbf{X}_i) =& \left\{E\left[\frac{h_{ij}^+}{\pi(A_j,\mathbf{X}_j)} \mid A_i = 1, \mathbf{X}_i\right] + E\left[\frac{h_{ij}^-}{\pi(A_j,\mathbf{X}_j)} \mid A_i = -1, \mathbf{X}_i\right]\right. \\
&\left. + E\left[\frac{h_{ij}^+}{\pi(A_j,\mathbf{X}_j)} \mid A_i = -1, \mathbf{X}_i\right] + E\left[\frac{h_{ij}^-}{\pi(A_j,\mathbf{X}_j)} \mid A_i = 1, \mathbf{X}_i\right]\right\} \\
=& \left\{E\left[\frac{|h_{ij}|}{\pi(A_j,\mathbf{X}_j)} \mid A_i = 1, \mathbf{X}_i\right] + E\left[\frac{|h_{ij}|}{\pi(A_j,\mathbf{X}_j)} \mid A_i = -1, \mathbf{X}_i\right]\right\} > 0.
\end{aligned}$$

Note that

$$2\tilde{p}(\mathbf{X}_i) - 1 = \left\{E\left[\frac{h_{ij}}{\pi(A_j,\mathbf{X}_j)} \mid A_i = 1, \mathbf{X}_i\right] - E\left[\frac{h_{ij}}{\pi(A_j,\mathbf{X}_j)} \mid A_i = -1, \mathbf{X}_i\right]\right\}\Big/ Z(\mathbf{X}_i),$$

and the Bayes rule $f^* = \text{sgn}\{2\tilde{p}(\mathbf{X}_i) - 1\} = \text{sgn}\{(2\tilde{p}(\mathbf{X}_i) - 1)Z(\mathbf{X}_i)\}$. By Theorem 2.31 of Steinwart and Christmann[3], for $-1 \le \dot{f} \le 1$,

$$R_\phi(\dot{f}) - R^*_\phi = \int |\dot{f}(\mathbf{X}_i) - f^*(\mathbf{X}_i)| \times \left| E\left[\frac{h_{ij}}{\pi(A_j, \mathbf{X}_j)} \mid A_i = 1, \mathbf{X}_i\right] - E\left[\frac{h_{ij}}{\pi(A_j, \mathbf{X}_j)} \mid A_i = -1, \mathbf{X}_i\right]\right| dP(\mathbf{X}_i)$$

Here, we need to introduce the geometric noise assumption to bound the excess ϕ-risk. Let $Q^h_{ij} = h_{ij}/\pi(A_j, \mathbf{X}_j)$, define

$$\eta(\mathbf{X}_i) = \frac{E[Q^h_{ij} \mid A_i = 1, \mathbf{X}_i = \mathbf{x}_i] - E[Q^h_{ij} \mid A_i = -1, \mathbf{X}_i = \mathbf{x}_i]}{2} + \frac{1}{2},$$

and the decision boundary is $2\eta(\mathbf{X}_i) - 1$, i.e., $f^* = \text{sgn}(2\eta(\mathbf{X}_i) - 1)$. Let $\mathcal{X}^+ = \{\mathbf{x} \in \mathcal{X} : 2\eta(\mathbf{x}) - 1 \ge 0\}$ and $\mathcal{X}^- = \{\mathbf{x} \in \mathcal{X} : 2\eta(\mathbf{x}) - 1 < 0\}$. A distance function to the boundary is defined as $\Delta(\mathbf{x}) = \tilde{d}(\mathbf{x}, \mathcal{X}^-)$, if $\mathbf{x} \in \mathcal{X}^+$; $\Delta(\mathbf{x}) = \tilde{d}(\mathbf{x}, \mathcal{X}^+)$ if $\mathbf{x} \in \mathcal{X}^-$, where $\tilde{d}(\mathbf{x}, \mathcal{X}^-)$ is the Euclidean distance from a point $\mathbf{x}$ to a set $\mathcal{X}^-$. The distribution of $(Y_i, A_i, \mathbf{X}_i)$ is said to have geometric nise component $0 < q < \infty$, if there exists a constant $C > 0$ s.t.

$$E\left[\exp\left(-\frac{\Delta(\mathbf{X}_i)^2}{t}\right)|2\eta(\mathbf{X}_i) - 1|\right] \le Ct^{qp/2}, t > 0.$$

According to the proof of theorem 2.7 in Steinwart and Scovel[2], we know that $|\dot{f}(\mathbf{X}_i) - f^*(\mathbf{X}_i)| \le 8e^{-\Delta(\mathbf{X}_i)^2/(2p\sigma_n^2)}$, and then

$$\begin{aligned} R_\phi(\dot{f}) - R^*_\phi &= E_{\mathbf{X}_i}[|2\eta(\mathbf{X}_i) - 1| \cdot |\dot{f}(\mathbf{X}_i) - f^*(\mathbf{X}_i)|] \\ &\le E\left[8\exp\left(\frac{-\Delta(\mathbf{X}_i)^2}{2p\sigma_n^2}\right)|2\eta(\mathbf{X}_i) - 1|\right] \le 8C(2p\sigma_n^2)^{pq/2} \end{aligned} \tag{6}$$

Combining the results in (5) and (6), we have

$$\inf_{f \in \mathcal{H}_{\sigma_n}} \{R_\phi(f) + \lambda_n \|f\|_k^2 - R^*_\phi\} \le c_p \sigma_n^p \lambda_n + 8C(2p)^{qp/2}\sigma_n^{qp}.$$

In particular, if we let $\sigma_n = \lambda_n^{-1/(qp+p)}$, the approximation error

$$\inf_{f \in \mathcal{H}_{\sigma_n}} \{R_\phi(f) + \lambda_n \|f\|_k^2 - R^*_\phi\} = O_p(\lambda_n^{q/(q+1)}). \tag{7}$$

Deriving the convergence rate of stochastic error (3) relies on the empirical process theory, which is not directly applicable to the CWL since its empirical risk function is not a summation of independent quantities for all subjects but involves the pair-wise contrast. To solve this problem, we use the idea of Hoeffding's decomposition[4], a commonly-used strategy in the U-statistics theory, and construct an intermediate empirical ϕ-risk function in the form of i.i.d. summation.

Consider the ϕ-risk function for $-1 \le f \le 1$,

$$\begin{aligned}
R_\phi(f) &= E\left[\frac{(1-\text{sgn}(h_{ij})A_i f(\mathbf{X}_i))|h_{ij}|}{\pi(A_i,\mathbf{X}_i)\pi(A_j,\mathbf{X}_j)}\right] \\
&= E\left[\frac{1-[h_{ij}/\pi(A_j,\mathbf{X}_j)]A_i f(\mathbf{X}_i)}{\pi(A_i,\mathbf{X}_i)}\right] + E\left[\frac{|h_{ij}|-\pi(A_j,\mathbf{X}_j)}{\pi(A_i,\mathbf{X}_i)\pi(A_j,\mathbf{X}_j)}\right] \\
&= E\left\{E\left[\frac{1-[h_{ij}/\pi(A_j,\mathbf{X}_j)]A_i f(\mathbf{X}_i)}{\pi(A_i,\mathbf{X}_i)} \mid Y_i, A_i, \mathbf{X}_i\right]\right\} + E\left[\frac{|h_{ij}|-\pi(A_j,\mathbf{X}_j)}{\pi(A_i,\mathbf{X}_i)\pi(A_j,\mathbf{X}_j)}\right] \\
&= E\left[\frac{1-E[h_{ij}/\pi(A_j,\mathbf{X}_j) \mid Y_i, A_i, \mathbf{X}_i]A_i f(\mathbf{X}_i)}{\pi(A_i,\mathbf{X}_i)}\right] + E\left[\frac{|h_{ij}|-\pi(A_j,\mathbf{X}_j)}{\pi(A_i,\mathbf{X}_i)\pi(A_j,\mathbf{X}_j)}\right]
\end{aligned}$$

Note that the second term above does not contain f, so we can omit is in further derivation. Let $C^h_{i,0} = E[h_{ij}/\pi(A_j,\mathbf{X}_j) \mid Y_i, A_i, \mathbf{X}_i]$ and $C^h_{i,0}$, $i = 1,\dots,n$, are i.i.d. statistics. Define

$$\hat{R}_{\phi,n}(f) = \frac{1}{n}\sum_{i=1}^n \phi(\text{sgn}(C^h_{i,n})A_i f(\mathbf{X}_i)) \cdot \frac{|C^h_{i,n}|}{\pi(A_i,\mathbf{X}_i)},$$

$$\tilde{R}_{\phi,n}(f) = \frac{1}{n}\sum_{i=1}^n \phi(\text{sgn}(C^h_{i,0})A_i f(\mathbf{X}_i)) \cdot \frac{|C^h_{i,0}|}{\pi(A_i,\mathbf{X}_i)},$$

where $\hat{R}_{\phi,n}(f)$ is the empirical ϕ-risk we used in the estimation and $\tilde{R}_{\phi,n}(f)$ is an intermediate empirical ϕ-risk in the form of i.i.d. summation. By the law of large numbers for i.i.d. random variables and U-statistics[4], we have

$$\tilde{R}_{\phi,n}(f) = R_\phi(f) + O_p(n^{-1/2}) \text{ and } \hat{R}_\phi(f) = R_{\phi,n}(f) + O_p(n^{-1/2}) \text{ for any } f \in \mathcal{H}_{\sigma_n}. \tag{8}$$

The corresponding estimated decision functions are:

$$\hat{f} = \arg\min_{f\in\mathcal{H}_{\sigma_n}} \hat{R}_{\phi,n}(f) + \lambda_n\|f\|_k^2, \quad \tilde{f} = \arg\min_{f\in\mathcal{H}_{\sigma_n}} \tilde{R}_{\phi,n}(f) + \lambda_n\|f\|_k^2.$$

Note that $C^h_{i,0}$ is not an observed quantity, thus $\tilde{f}$ is only used as a theoretical device. By the law of large numbers, it is easy to find that $C^h_{i,n} = C^h_{i,o} + O_p(n^{-1/2})$. Given Y_i satisfying $P[Y_i = E(Y_i)] = 0$, we have $\hat{R}_{\phi,n}(f) - \tilde{R}_{\phi,n}(f) = O_p(n^{-1/2})$ for any $f \in \mathcal{H}_{\sigma_n}$, and then

$$\begin{aligned}
&\inf_{f\in\mathcal{H}_{\sigma_n}}\{\hat{R}_{\phi,n}(f)+\lambda_n\|f\|_k^2\} - \inf_{f\in\mathcal{H}_{\sigma_n}}\{\tilde{R}_{\phi,n}(f)+\lambda_n\|f\|_k^2\} \\
=&\hat{R}_{\phi,n}(\hat{f}_n)+\lambda_n\|\hat{f}_n\|_k^2 - \tilde{R}_{\phi,n}(\tilde{f}_n)+\lambda_n\|\tilde{f}_n\|_k^2\} = O_p(n^{-1/2}).
\end{aligned} \tag{9}$$

The stochastic error can be further separated as follows:

$$\begin{aligned}
&R_\phi(\hat{f}_n)+\lambda_n\|\hat{f}_n\|_k^2 - \inf_{f\in\mathcal{H}_{\sigma_n}}\{R_\phi(f)+\lambda_n\|f\|_k^2\} \\
=&R_\phi(\hat{f}_n)+\lambda_n\|\hat{f}_n\|_k^2 - R_\phi(\tilde{f}_n)+\lambda_n\|\tilde{f}_n\|_k^2 + R_\phi(\tilde{f}_n)+\lambda_n\|\tilde{f}_n\|_k^2 - \inf_{f\in\mathcal{H}_{\sigma_n}}\{R_\phi(f)+\lambda_n\|f\|_k^2\}
\end{aligned}$$

For the second line above, combining (8) and (9), we have

$$R_\phi(\hat{f}_n) + \lambda_n\|\hat{f}_n\|_k^2 - R_\phi(\tilde{f}_n) + \lambda_n\|\tilde{f}_n\|_k^2$$
$$=\hat{R}_{\phi,n}(\hat{f}_n) + \lambda_n\|\hat{f}_n\|_k^2 - \tilde{R}_{\phi,n}(\tilde{f}_n) + \lambda_n\|\tilde{f}_n\|_k^2 + O_p(n^{-1/2}) = O_p(n^{-1/2}) \quad (10)$$

Since $\tilde{f}_n$ is an estimator that minimizes an empirical ϕ-risk in the form of i.i.d. summation and the ϕ-risk is identical to the one in the outcome weighted learning except for the different weights, we can follow the proof of Theorem 3.4 in Zhao et al.[5] to obtain the convergence rate. For $q > 0$, $\delta > 0$, $0 < \nu < 2$, and constants C_1 and C_2 depending on them,

$$R_\phi(\tilde{f}_n) + \lambda_n\|\tilde{f}_n\|_k^2 - \inf_{f\in\mathcal{H}_{\sigma_n}}\{R_\phi(f) + \lambda_n\|f\|_k^2\} = C_1(\lambda_n)^{-\frac{2}{2+\nu}+\frac{(2-\nu)(1+\delta)}{(2+\nu)(1+q)}} n^{-\frac{2}{2+\nu}} + C_2(n\lambda_n)^{-1}.$$

When $\lambda_n = n^{\frac{-2(1+q)}{(4+\nu)q+2+(2-\nu)(1+\delta)}}$, combine above equation with the convergence rate for approximation error (7) and the difference between two empirical risk functions (10) to obtain the optimal convergence rate:

$$R(\hat{f}_n) - R^* = O_p(n^{-\frac{2q}{(4+\nu)q+2+(2-\nu)(1+\delta)}}).$$

Web Appendix 2 | ADDITIONAL NUMERICAL RESULTS

Web Appendix 2.1 | Implementation details

Recall that the OTR under CWL is estimated as:

$$\hat{f}_n = \arg\min_{f\in\mathcal{H}_k} \frac{1}{n}\sum_{i=1}^n \phi(\widetilde{A}_i f(\mathbf{X}_i)) \cdot \frac{|C_{i,n}^h|}{\pi(A_i,\mathbf{X}_i)} + \lambda_n\|f\|_k^2,$$

where $\mathcal{H}_k$ is the reproducing kernel Hilbert space and $\|\cdot\|_k$ is the norm induced by a given kernel function $k(\cdot,\cdot)$. Above function can be view as an objective function in the weighted support vector machine (SVM) using hinge loss $\phi(\cdot)$ with weight $|C_{i,n}^h|/\pi(A_i,\mathbf{X}_i)$ and class label $\widetilde{A}_i$. Define the decision function as:

$$f(\mathbf{x}) = \sum_{i=1}^n \alpha_i k(\mathbf{x},\mathbf{x}_i) + \alpha_0,$$

where $\boldsymbol{\alpha} = (\alpha_1,\dots,\alpha_n) \in \mathbb{R}^n$. The minimization problem can be written as follows:

$$\min \frac{1}{2}\boldsymbol{\alpha}^T\mathbf{K}\boldsymbol{\alpha} + s_n\sum_{i=1}^n \frac{|C_{i,n}^h|}{\pi(A_i,\mathbf{X}_i)}\xi_i$$
$$\text{subject to } \tilde{A}_i\left[\sum_{i=1}^n \alpha_i k(\mathbf{x},\mathbf{x}_i) + \alpha_0\right] \geq 1-\xi_i,\ \xi_i \geq 0,\ \text{for } i = 1,\dots,n,$$

where $\mathbf{K} = [k(\mathbf{x}_a,\mathbf{x}_b)]$ is an $n\times n$ matrix for $a,b = 1,\dots,n$, s_n is a tuning parameter depending on λ_n, and ξ_i is a slack variable that allows a certain amount of misclassification. By the method of Lagrange multipliers, the optimization problem turns out to

be a dual problem:

$$\max_{\boldsymbol{\alpha}} \sum_{i=1}^{n} \alpha_i - \frac{1}{2}\sum_{i=1}^{n}\sum_{j=1}^{n} \alpha_i \alpha_j \widetilde{A}_i \widetilde{A}_j k(\mathbf{X}_i, \mathbf{X}_j),$$
$$\text{subject to } 0 \le \alpha_i \le \frac{\kappa_n |C_i^h|}{\pi(A_i, \mathbf{X}_i)},\ i = 1, \ldots, n,$$
$$\sum_{i=1}^{n} \alpha_i A_i = 0,$$

where κ_n is a tuning parameter determined by λ_n. In particular, if f is constraint to be a linear function, we will replace $k(\cdot,\cdot)$ by inner-product $\langle \cdot, \cdot \rangle$. The estimated decision function is $\hat{f}_n(\mathbf{x}) = \sum_{i=1}^{n} \hat{\alpha}_i \widetilde{A}_i k(\mathbf{x}, \mathbf{x}_i) + \hat{\alpha}_0$, where $\hat{\alpha}_0$ can be solved by using the margin points subject to the Karush-Kuhn-Tucker conditions[6], and the estimated decision rule is given by $\hat{d}(\mathbf{x}) = 2I(\hat{f}_n(\mathbf{x}) \ge 0) - 1$. Many quadratic programming algorithm can be implemented to train the weighted SVM. We choose to use the `wsvm` function in the R package named `WeightSVM`[7].

Web Appendix 2.2 | Additional simulation studies

The selection of covariates potentially influences the performance of SVM-based OTR estimation methods using nonlinear kernel[8]. We explore the effect of unrelated covariates on OTR estimation. In particular, we increase the dimension of covariates from 5 to 10 by introducing 5 additional unrelated covariates under the nonlinear boundary scenarios with continuous outcomes. Other parameter settings are identical to those in the main article. According to Figure 1, the value of all methods declines after introducing the unrelated covariates, which is not surprising given the moderate sample sizes in our simulations. Nevertheless, CWL methods still outperform the existing methods, and the margins increase with sample size.

The numerical summary of the results for continuous and ordinal outcomes under various scenarios can be found in Table 1 and 2, respectively.

We conduct additional simulations with different parameter settings for continuous outcomes. Specifically, we simulate continuous outcomes with outliers with a smaller magnitude of $\mu_O = 10$ and generate less skewed continuous outcomes with $\sigma_{\log} = 1$. Under these settings, CWL with win indicator and truncated difference contrasts are still the top performers although their margins over the other methods decrease, largely due to the improved values in the other methods (Figure 2.) We also consider an additional implementation of residual weighted learning. In lieu of fitting the linear regression model to compute residuals, we use the random forest to fit the data and compute the residuals based on predicted results. The results suggest no significant difference between using linear regression or random forest even if the linear regression model with linear main effect is misspecified under nonlinear settings. This implies that the model fitting part in residual weighted learning mainly contributes to reducing the variation in the outcome related to covariates. It is not necessary to have a correct specification of the main effect model.

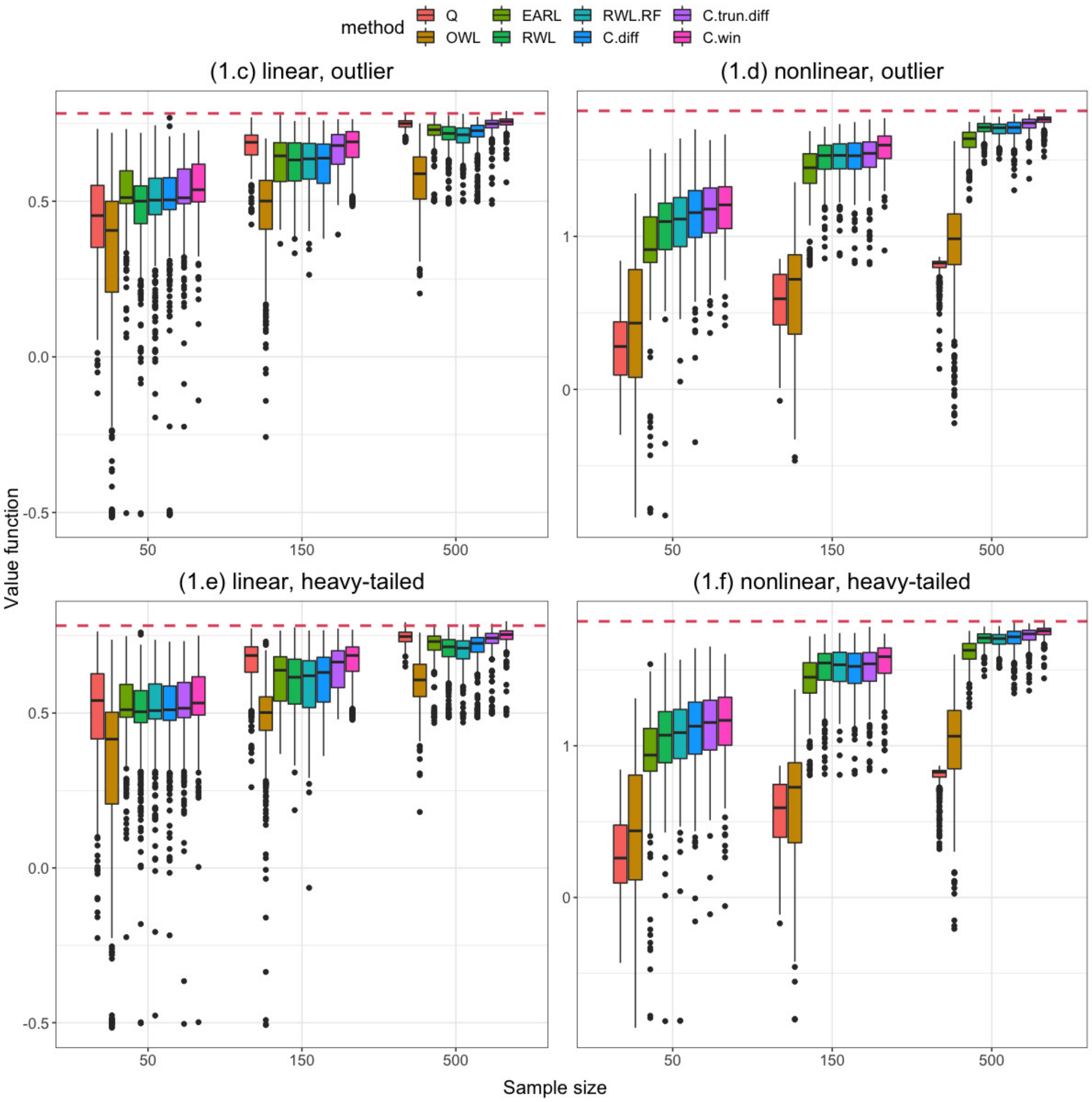

FIGURE 1 The comparison of simulations results for the number of covariates $p = 5$ and $p = 10$ in three scenarios with a nonlinear decision boundary. The seven methods we compare (from left to right in plots) are: Q-learning (Q), outcome weighted learning (OWL), efficient augmentation and relaxation learning (EARL), residual weighted learning using linear regression residual (RWL) and random forest regression residual (RWL.RF), and contrast weighted learning using difference contrast (C.diff), truncated difference contrast (C.trun.diff), and win indicator contrast(C.win). The dashed horizontal line represents the theoretical optimal value.

In all the weighted learning methods, the propensity score is estimated before OTR estimation. We evaluate the effect of misspecified propensity score model on the performance of weight learning methods. Two types of misspecification are considered. In scenario 1, the true propensity score model is assumed to be a logistic model logit$[P(A_i = 1 \mid \mathbf{X}_i)] = X_{i1}X_{i3}+X_{i5}^2,\ i = 1, ..., n$ whereas the fitted model is misspecified as a logistic model logit$[P(A_i = 1 \mid \mathbf{X}_i)] = X_{i1} + X_{i3} + X_{i5}$. In scenario 2, the true propensity score model is set to be a logistic model logit$[P(A_i = 1 \mid \mathbf{X}_i)] = 0.2(X_{i1}+X_{i2}+X_{i3}+X_{i4}+X_{i5}),\ i = 1, ..., n$ whereas the fitted model is misspecified as a logistic model logit$[P(A_i = 1 \mid \mathbf{X}_i)] = 0.2(X_{i1} + X_{i3} + X_{i5})$. Other settings are identical to

TABLE 1 Mean (SD) of the value functions in 400 replications for the data with continuous outcomes under 6 scenarios (1.a)-(1.f); $\mu_O = 15$ in (1.c),(1.d); $\sigma_{\log} = 1.5$ in (1.e),(1.f)

Scenario	n	Q	OWL	EARL	RWL	C.diff	C.trun.diff	C.win
(1.a)	50	0.651	0.335	0.568	0.577	0.566	0.565	0.56
linear,		(0.075)	(0.264)	(0.093)	(0.085)	(0.085)	(0.084)	(0.102)
normal	150	0.75	0.484	0.701	0.703	0.687	0.69	0.68
		(0.016)	(0.179)	(0.053)	(0.051)	(0.068)	(0.064)	(0.075)
	500	0.773	0.599	0.76	0.754	0.758	0.758	0.756
		(0.007)	(0.082)	(0.012)	(0.017)	(0.019)	(0.018)	(0.023)
(1.b)	50	0.348	0.405	1.065	1.201	1.239	1.236	1.239
nonlinear,		(0.258)	(0.453)	(0.248)	(0.203)	(0.205)	(0.221)	(0.21)
normal	150	0.634	0.654	1.566	1.642	1.642	1.642	1.639
		(0.207)	(0.366)	(0.1)	(0.07)	(0.09)	(0.085)	(0.086)
	500	0.818	1.022	1.657	1.745	1.768	1.768	1.77
		(0.049)	(0.302)	(0.055)	(0.023)	(0.029)	(0.03)	(0.026)
(1.c)	50	0.33	0.291	0.501	0.417	0.465	0.529	0.548
linear,		(0.192)	(0.274)	(0.159)	(0.198)	(0.205)	(0.118)	(0.103)
outlier	150	0.616	0.437	0.587	0.575	0.59	0.659	0.677
		(0.074)	(0.185)	(0.087)	(0.093)	(0.087)	(0.075)	(0.072)
	500	0.72	0.559	0.671	0.661	0.672	0.737	0.751
		(0.03)	(0.105)	(0.074)	(0.076)	(0.07)	(0.047)	(0.028)
(1.d)	50	0.236	0.388	0.887	1.01	1.108	1.131	1.175
nonlinear,		(0.246)	(0.445)	(0.335)	(0.258)	(0.238)	(0.224)	(0.222)
outlier	150	0.484	0.626	1.331	1.445	1.467	1.506	1.58
		(0.239)	(0.353)	(0.199)	(0.154)	(0.159)	(0.146)	(0.119)
	500	0.742	0.945	1.58	1.658	1.641	1.711	1.755
		(0.139)	(0.309)	(0.092)	(0.066)	(0.081)	(0.059)	(0.037)
(1.e)	50	0.224	0.228	0.325	0.279	0.338	0.404	0.476
linear,		(0.239)	(0.3)	(0.272)	(0.297)	(0.254)	(0.208)	(0.163)
heavy-tailed	150	0.367	0.357	0.439	0.402	0.442	0.562	0.616
		(0.2)	(0.217)	(0.205)	(0.243)	(0.18)	(0.095)	(0.092)
	500	0.501	0.464	0.51	0.503	0.512	0.675	0.723
		(0.186)	(0.172)	(0.168)	(0.167)	(0.161)	(0.094)	(0.075)
(1.f)	50	0.16	0.338	0.539	0.759	0.823	0.915	0.988
nonlinear,		(0.254)	(0.449)	(0.547)	(0.389)	(0.333)	(0.293)	(0.278)
heavy-tailed	150	0.279	0.519	0.96	1.099	1.115	1.247	1.389
		(0.251)	(0.376)	(0.389)	(0.284)	(0.282)	(0.221)	(0.2)
	500	0.461	0.792	1.307	1.388	1.377	1.568	1.689
		(0.26)	(0.342)	(0.223)	(0.194)	(0.205)	(0.143)	(0.088)

the scenario (1.c) in main article. The results suggest that under both misspcification scenarios, the weighted learning methods are fairly robust to the misspecification of propensity score models (Figure 3).

TABLE 2 Mean (SD) of the value functions in 400 replications for the data with evenly or unevenly distributed ordinal outcome under scenarios (2.a) and (2.b)

Scenario	n	Q	OWL	EARL	RWL	C.diff	C.trun.diff	C.win
(2.a)	50	1.782	1.684	1.765	1.783	1.799	1.801	1.785
linear,		(0.061)	(0.155)	(0.111)	(0.073)	(0.062)	(0.06)	(0.074)
uneven	150	1.851	1.772	1.815	1.826	1.835	1.835	1.832
		(0.016)	(0.099)	(0.046)	(0.025)	(0.02)	(0.02)	(0.021)
	500	1.868	1.818	1.835	1.847	1.849	1.85	1.85
		(0.004)	(0.036)	(0.007)	(0.013)	(0.015)	(0.015)	(0.014)
(2.b)	50	1.828	1.859	1.91	2.051	2.062	2.067	2.05
nonlinear,		(0.074)	(0.152)	(0.145)	(0.093)	(0.088)	(0.08)	(0.086)
uneven	150	1.902	1.978	2.005	2.173	2.189	2.191	2.163
		(0.075)	(0.113)	(0.09)	(0.076)	(0.08)	(0.081)	(0.069)
	500	1.984	2.149	2.133	2.265	2.314	2.312	2.293
		(0.038)	(0.088)	(0.061)	(0.035)	(0.027)	(0.03)	(0.039)
(2.a)	50	2.693	2.541	2.615	2.686	2.689	2.695	2.703
linear,		(0.077)	(0.154)	(0.129)	(0.084)	(0.085)	(0.075)	(0.06)
even	150	2.796	2.625	2.693	2.755	2.765	2.764	2.765
		(0.023)	(0.126)	(0.062)	(0.039)	(0.04)	(0.04)	(0.037)
	500	2.833	2.687	2.726	2.806	2.809	2.808	2.809
		(0.007)	(0.063)	(0.029)	(0.029)	(0.035)	(0.037)	(0.034)
(2.b)	50	2.586	2.651	2.673	2.881	2.923	2.919	2.885
nonlinear,		(0.084)	(0.155)	(0.161)	(0.103)	(0.098)	(0.098)	(0.101)
even	150	2.67	2.767	2.796	3.061	3.107	3.113	3.08
		(0.091)	(0.141)	(0.114)	(0.099)	(0.078)	(0.076)	(0.08)
	500	2.761	2.986	2.988	3.176	3.221	3.224	3.209
		(0.039)	(0.117)	(0.093)	(0.035)	(0.029)	(0.024)	(0.035)

Web Appendix 2.3 | Real data analysis

In this subsection, we provide more details about the data in the ACE-IPF[9] and ORCHID[10] trials. The endpoint in ACE-IPF trial we analyze is the percentage changes in the percentage change from baseline in forced vital capacity (FVC). From the histograms in Figure 4, we can see that the distribution of outcomes presents slight skewness. Using the skewness measure defined as $(1-1/n)^{3/2}m_3/m_2^{3/2}$ [11], where m_r are the rth sample central moments, we have that the overall distribution has a -0.56 skewness, which suggests slight left skewness. One patient had a 40% decrease and is a potential outlier in the data. For the ordinal outcomes in ORCHID trial, the bar plots Figure 4 illustrates that most inpatients tend to have a mild situation after two weeks hospitalization regardless of their treatment assignments.

In addition to the boxplots, we present in the main article, the mean and standard deviation of the testing value functions in the 100 replicated cross-validations are reported in Table 3. Due to the small treatment effect size and potential interference from other factors in the real world, the superiority of CWL is not as obvious as in the simulation studies. Nevertheless, CWL still exhibits some advantages over the existing methods, especially when a Gaussian kernel is used.

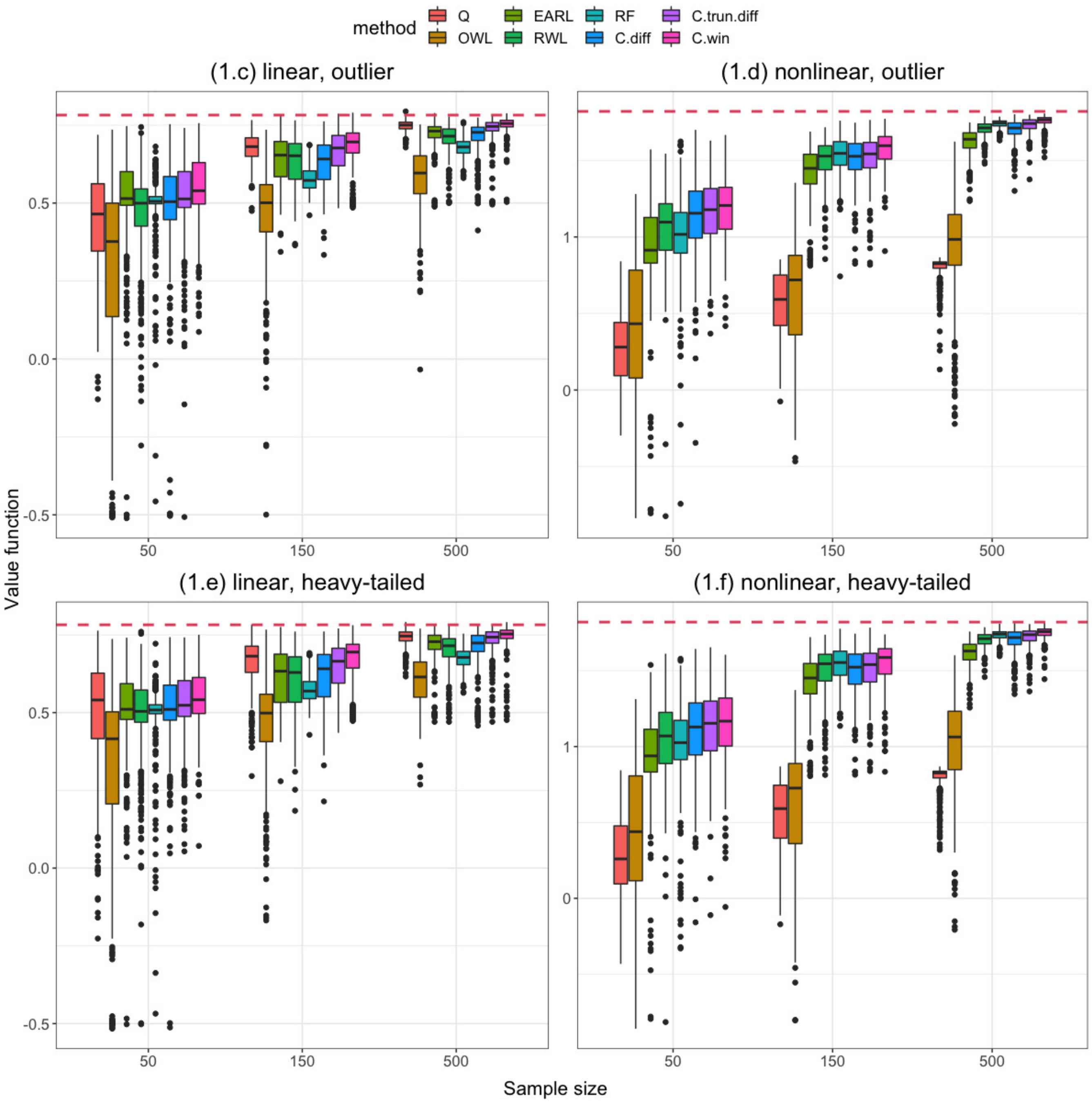

FIGURE 2 Simulation results for additional parameter settings: scenario (1.c) and (1.d) with outlier $\mu_O = 10$; scenario (1.e) and (1.f) with $\sigma_{\log} = 1$. Scenario (1.a) is the normal settings, which works as a reference. The dashed horizontal line represents the theoretical optimal value.

After performing the cross-validation, we implement each method to estimate a decision rule using all the observed data and summarize the characteristics of the control and treatment groups under the estimated rules. Table 4 presents the summary of covariates for seven estimated rules using Gaussian kernel and the observed assignments in ACE-IPF trials. We choose to explore the decision rules with nonlinear boundaries because they overall have a better performance than linear decision rules in the

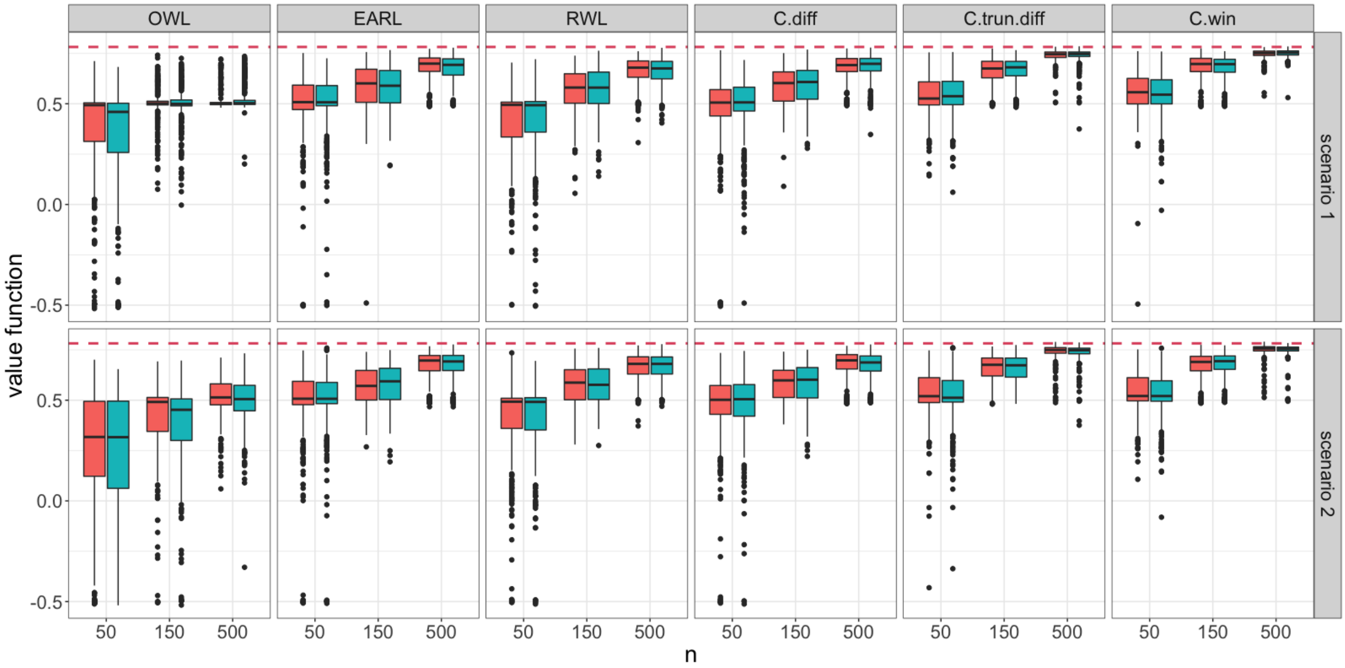

FIGURE 3 Testing value functions for weighted learning methods using correctly specified (red) and misspecified (blue) propensity score models. The dashed horizontal line represents the theoretical optimal value.

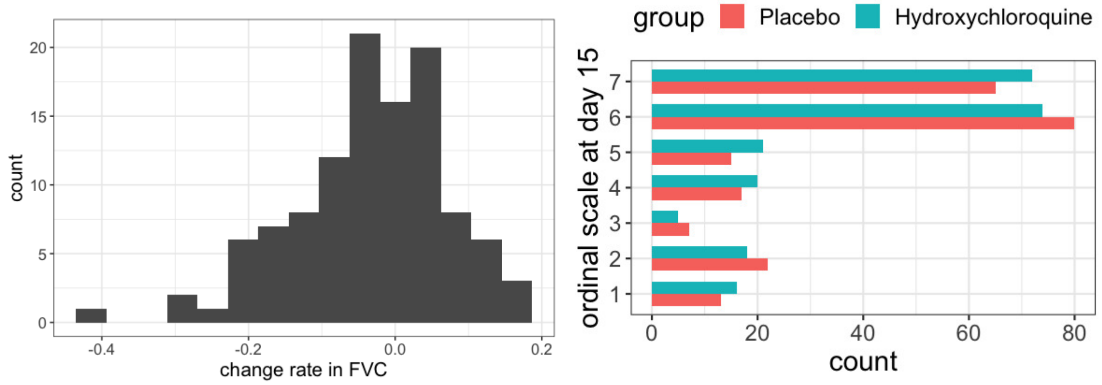

FIGURE 4 Distributions of the outcomes in ACE-IPF trial (left) and ORCHID (right).

cross-validation. Since the nonlinear decision boundary is not easy to interpret directly, we summarize the profiles of estimated treatment and control groups to see which covariate plays a role in decision making. In ACE trial, the proportions of treatment and control under the estimated rules are still close 1:1. The only exception is that the rule obtained by Q-learning assigns 73% subjects to control group. Based on our cross-validation results, Q-learning has the lowest validating value function. Although the features of observed control and treatment groups are similar due to randomization, several estimated rules tend to assign older patients or patients with a large lung capacity to control groups.

TABLE 3 Mean (SD) of value functions in 100 replicated cross-validations for the data from ORCHID and ACE-IPF trials

trial	kernel	Q	OWL	EARL	RWL	C.diff	C.trun.diff	C.win
ACE-IPF	linear	-0.105	-0.079	-0.102	-0.101	-0.097	-0.097	-0.095
		(0.026)	(0.02)	(0.024)	(0.024)	(0.025)	(0.024)	(0.025)
	Gaussian	-0.105	-0.075	-0.071	-0.054	-0.049	-0.049	-0.047
		(0.027)	(0.024)	(0.028)	(0.029)	(0.025)	(0.023)	(0.023)
ORCHID	linear	5.315	5.258	5.201	5.1	5.231	5.227	5.17
		(0.117)	(0.067)	(0.067)	(0.219)	(0.086)	(0.085)	(0.071)
	Gaussian	5.315	5.232	5.227	5.14	5.276	5.27	5.294
		(0.117)	(0.08)	(0.078)	(0.266)	(0.088)	(0.091)	(0.08)

TABLE 4 Summary of subject characteristics of treatment and control groups under estimated rule with Gaussian kernel (or observed assignment) in ACE-IPF trial. 'LGVTLC' is the total lung capacity.

method	group	proportion	LGVTLC	age	female%	smoke status		
						never	current	past
Q	control	0.73	4.018	68.7	0.21	0.222	0.778	0
	treatment	0.27	3.086	61.1	0.5	0.333	0.467	0.2
OWL	control	0.477	3.877	66.9	0.245	0.17	0.755	0.075
	treatment	0.523	3.665	66.4	0.328	0.328	0.638	0.034
EARL	control	0.676	3.819	67.7	0.253	0.253	0.72	0.027
	treatment	0.324	3.655	64.5	0.361	0.25	0.639	0.111
RWL	control	0.631	3.85	67.6	0.243	0.243	0.714	0.043
	treatment	0.369	3.622	64.9	0.366	0.268	0.659	0.073
C.diff	control	0.568	3.911	66.7	0.27	0.238	0.714	0.048
	treatment	0.432	3.576	66.5	0.312	0.271	0.667	0.062
C.trun.diff	control	0.613	3.871	67.7	0.265	0.235	0.735	0.029
	treatment	0.387	3.601	65	0.326	0.279	0.628	0.093
C.win	control	0.55	3.943	67.5	0.262	0.23	0.738	0.033
	treatment	0.45	3.55	65.6	0.32	0.28	0.64	0.08
observed	control	0.514	3.819	66.6	0.228	0.193	0.737	0.07
	treatment	0.486	3.71	66.7	0.352	0.315	0.648	0.037

References

1. Bartlett PL, Jordan MI, McAuliffe JD. Convexity, classification, and risk bounds. *Journal of the American Statistical Association* 2006; 101(473): 138–156.

2. Steinwart I, Scovel C. Fast rates for support vector machines using Gaussian kernels. *The Annals of Statistics* 2007; 35(2): 575–607.

3. Steinwart I, Christmann A. *Support vector machines*. Springer Science & Business Media . 2008.

4. Hoeffding W. A Class of Statistics with Asymptotically Normal Distribution. *The Annals of Mathematical Statistics* 1948; 19(3): 293–325.

5. Zhao Y, Zeng D, Rush AJ, Kosorok MR. Estimating individualized treatment rules using outcome weighted learning. *Journal of the American Statistical Association* 2012; 107(499): 1106–1118.

6. Friedman J, Hastie T, Tibshirani R, others . *The elements of statistical learning*. Springer series in statistics New York . 2001.

7. Xu T, Chang CC, Lin CC, et al. WeightSVM: Subject Weighted Support Vector Machines. https://CRAN.R-project.org/package=WeightSVM; 2020.

8. Zhou X, Mayer-Hamblett N, Khan U, Kosorok MR. Residual weighted learning for estimating individualized treatment rules. *Journal of the American Statistical Association* 2017; 112(517): 169–187.

9. Noth I, Anstrom KJ, Calvert SB, et al. A placebo-controlled randomized trial of warfarin in idiopathic pulmonary fibrosis. *American journal of respiratory and critical care medicine* 2012; 186(1): 88–95.

10. Self WH, Semler MW, Leither LM, et al. Effect of hydroxychloroquine on clinical status at 14 days in hospitalized patients with COVID-19: a randomized clinical trial. *Jama* 2020; 324(21): 2165–2176.

11. Joanes DN, Gill CA. Comparing measures of sample skewness and kurtosis. *Journal of the Royal Statistical Society: Series D (The Statistician)* 1998; 47(1): 183–189.

□
